# Effect of Gamma Radiation on the Chemical Structure and Physical Properties of Poly(butylene terephthalate) (PBT)

**DOI:** 10.3390/polym18172123

**Published:** 2026-08-31

**Authors:** Daniel Marcos-Rios, Guillermina Burillo, Rodrigo Navarro, Ángel Marcos-Fernández

**Affiliations:** 1 Institute of Polymer Science and Technology (ICTP-CSIC), Juan de la Cierva, 3, 28006 Madrid, Spain; d.marcos@ictp.csic.es (D.M.-R.); rnavarro@ictp.csic.es (R.N.); 2 Instituto de Ciencias Nucleares, Universidad Nacional Autónoma de México, Circuito Exterior s/n, Ciudad Universitaria, Delegación Coyoacán, Mexico City C.P. 04510, Mexico; burillo@nucleares.unam.mx

**Keywords:** PBT, gamma, chemical changes, chain scission, crosslinking

## Abstract

This study presents the effect of gamma rays of up to 2000 kGy on the chemical structure and the physical properties of a poly(butylene terephthalate) (PBT). PBT is an important engineering polymer, tougher than PET, that can be upgraded by radiation crosslinking with the addition of crosslinkers. Previous studies found in the literature provided very limited data about the effect of ionizing radiation on pure PBT. No gel was produced, and the changes in molecular weight with the increase in dose were in agreement with the results found in the literature, with a decrease until 1000 kGy dose when chain scission predominated and a slight increase at higher doses when crosslinking became significant. Proton NMR analysis was used for the first time in PBT to determine the changes in chemical species, mainly the appearance of terminal butyl groups and aromatic carboxylic groups coming from the rupture of the ester linkage. Both thermal and mechanical properties were explained by the scission of the chains in the amorphous phase and at the boundaries of the crystallites, and the crosslinking in the amorphous phase. The thermal parameter most affected by irradiation was the crystallization temperature, which increased when chain scission was predominant up to a 1000 kGy dose and decreased at higher doses when crosslinking became significant. Stress and strain at break suffered a continuous decrease with dose until PBT became fragile at high dose, with a faster decrease in strain at break from the 1000 kGy dose, when crosslinking became significant.

## 1. Introduction

When a polymer is subjected to ionizing radiation (e-beam, gamma), the energy is absorbed and transferred to the electrons in the material, leading to the ionization and excitation of atoms and molecules. This primary interaction results in the formation of free radicals, which are highly reactive and initiate a series of secondary reactions within the polymer matrix. The overall effect of ionizing radiation on a polymer depends on several variables, including the polymer’s chemical structure, molecular weight, degree of crystallinity, presence of additives or oxygen, and the irradiation conditions such as dose, dose rate, and atmosphere [1,2,3].

Ionizing radiation can produce two main events on a polymeric chain: chain scission and crosslinking. Chain scission leads to a decrease in molecular weight and therefore a deterioration of the polymer’s mechanical properties. Crosslinking, with the ultimate formation of a three-dimensional network, significantly improves the material’s mechanical strength, solvent resistance, stiffness, thermal properties, and thermal stability. Ionizing radiation is used to manufacture many articles when crosslinking is the main event produced in the polymeric chain, such as shrinkable tubing and tapes, wire and cable coatings, and partial crosslinking of automobile tire plies, among others [4].

Poly(butylene terephthalate) (PBT) is an aromatic semicrystalline engineering thermoplastic polymer, tougher than PET, with a wide range of applications in automotive, electrical and electronics, medical, and many more. Its market size in 2025 was 1.62 million tons, with more than 68% being modified PBT (typically reinforced PBT with glass fibers) [5].

PBT requires the addition of additives prior to processing in order that crosslinking reactions can be boosted during the irradiation; thus, it can be upgraded to approach the properties of high-performance polymers, especially a higher thermal stability with HDT > 150 °C [6,7]. In the early 90s, PBT manufacturers already patented the incorporation of reactive components that initiate crosslinking during the radiation process, offering processors a new material with the processing characteristics of standard PBT [6].

The first studies on the effect of ionizing radiation in aromatic polyesters started in the 60s to determine the durability of PET film in satellites when exposed to ionizing radiation in a near-Earth environment, and expanded later to other aromatic polyesters with variations in the polymeric chain [8].

Studies on the effect of ionizing radiation in PBT are scarce, and most of them are on PBT with crosslinkers, additives and fillers [6,7,9,10,11,12,13,14,15,16,17,18], and only a few studies have been carried out in pure PBT irradiated with gamma radiation [8,19,20,21,22], electron beam [23,24,25] and heavy ions [26]. Of these studies, measurement of the physical properties is rather uncommon, with limited results on the changes with dose in molecular weight, measured by SEC or viscosimetry, up to a dose of 10,000 kGy [8,21,22], on thermal properties up to a dose of 1100 kGy [8,21,26], and only one mention of changes in mechanical properties [27]. In the case of molecular weight changes, different behavior is described among the literature works, and a defined trend cannot be asserted. For the thermal properties, not all parameters are measured in the literature, and the changes in some of these parameters (Tg, crystallinity) rely on the data of a single report [21].

Thus, there is an evident lack of information related to the effect of ionizing radiation on the physical properties of PBT. We present a detailed study on the effect of the irradiation of PBT with gamma rays within a wide range of doses, up to 2000 kGy, in order to establish clear relationships for the thermal and mechanical properties. Furthermore, we have used NMR for the first time to identify the species generated by exposure of PBT to ionizing radiation, relate them to the stability of the different bonds in PBT, and propose a mechanism for the chain scission of the PBT polymer chains. These results provide a fundamental framework for predicting the behavior of this polymer when subjected to high-energy radiation.

## 2. Experimental

### 2.1. Materials

Poly(butylene terephthalate) (PBT) in the form of white pellets was provided by BASF SE (Ludwigshafen am Rhein, Germany) with the name Ultradur^®^ B6550. This high viscosity grade had a Melt Volume Rate (250 °C, 2.16 kg) of 8.9 cm^3^·10 min^−1^, and the following nominal properties: melting temperature of 223 °C (DSC, 250 °C·min^−1^); tensile Modulus 2400 MPa; tensile strain greater than 50%; melt temperature range, injection-molding 250–275 °C; mold temperature range, injection-molding 40–80 °C [28].

### 2.2. Sample Preparation

PBT was processed by injection using a Boy XS injection machine (Dr. Boy GmbH & Co, Neustadt-Fernthal, Germany). Pellets were previously dried at 140 °C for 2 h. in a hot dryer CTP 25 (Centrotecnica, Barcelona, Spain). Injected samples were obtained at a molding temperature of 265 °C (two heating zones, both at 265 °C) and a mold temperature of 75 °C. Injection pressure was 120 bar, injection speed 65 mm·s^−1^, clamping pressure 50 bar, holding time 5 s, and cooling time 20 s. Injected samples were rectangular plates with three different thicknesses: 1, 1.5, and 2 mm, as seen in Figure S1-left in the Supplementary Material. Test specimens for characterization were obtained from the injected plates (see Figure S1-right in the Supplementary Material for tensile specimens). Two sets of samples were injected: unirradiated pellets and irradiated pellets.

### 2.3. Gamma Irradiation

Two sets of samples were irradiated: pellets and testing specimens cut from injected PBT.

Pellets were introduced in polyethylene bags and sealed in air, whereas type 5B dumbbell test pieces (according to ISO 527-2) [29] for thermal and mechanical properties were cut from the unirradiated PBT sheet, introduced into 9 cm × 6 cm polyethylene bags, and sealed in air.

Pellets and test pieces were irradiated in air at room temperature with a ^60^Co–source (Gammabeam 651-PT, Nordion International Inc., Ottawa, ON, Canada) installed at the Instituto de Ciencias Nucleares of UNAM, México, with an activity of 63,000 Ci at a dose rate of 12.31 and 8.49 kGy h^−1^ for pellets and test specimens respectively, and doses from 100 to 2000 (100, 500, 1000, 1200, 1500, and 2000) kGy for pellets and from 10 to 2000 (10, 20, 30, 50, 100, 200, 500, 700, 1000, 1200, 1500 and 2000) kGy for dumbbell pieces. The determination of the dose rate for the gamma ray source was carried out with a modified Fricke (ferrous sulfate dosimeter modified by the addition of cupric sulfate) [30]. Once the absorbed dose was determined by the Fricke dosimeter, the absorbed dose in the polymer, referred to as the dose in this work, was calculated.

### 2.4. Characterization Methods

Viscosity measurements in solution. Inherent viscosity (η_inh_ in dL·g^−1^) was measured at 30 °C in a hexafluoroisopropanol (HFIP; Fluorochem, Hadfield, UK) solution at a concentration of 0.5 g·dL^−1^ with a Ubbelohde Viscometer (capillary #1) using the following equation:

$$\eta_{inh}=2\ln\left(\frac{t}{t_{0}}\right)$$

where *t*_0_ is the time to flow through the capillary for the neat solvent, and *t* is the time to flow for the sample solution. Flow times were measured at least seven times, with a calculated error for the mean value of less than 1%.

Melt viscosity measurements. Viscosity of the melt was measured at 230 °C in a Rubber Process Analyzer instrument (Alpha Technologies, Hudson, OH, USA). Two discs of 3.6 cm diameter, cut from the 2 mm thick injected plates from pellets irradiated at 0, 500, 1000, 1200, 1500, and 2000 kGy, weighing approximately 5.5 g, were placed in the chamber and heated at 230 °C for 30 min. Viscosity of the melt was measured at 0.05, 0.1, 0.21, 0.44, 0.90, 1.84, 3.80, 7.66, 16.34, and 33.33 Hz frequencies with a fixed strain of 7% four times on the same sample, with a 5-min delay between the measurements. The dispersion of the results is given as the standard deviation.

Nuclear Magnetic Resonance (NMR). Solution proton NMR spectra were recorded at room temperature on a Bruker Avance III HD instrument (Bruker BioSpin, Billerica, MA, USA) working at 400 MHz, using deuterated chloroform (CDCl_3_; Sigma-Aldrich, Steinheim, Germany) as the solvent with some drops of HFIP, and trifluoroacetic anhydride (TFA; TCI Europe, Zwijndrecht, Belgium) as a derivatizing agent. Spectra were referenced to the residual solvent signal at 7.26 ppm. Approximately 10 mg of the polymer was dissolved in 0.8 mL of deuterated solvent.

Differential Scanning Calorimetry (DSC). The thermal transitions of the samples were analyzed by DSC on a Perkin Elmer DSC 7 calorimeter (Perkin Elmer, Shelton, CT, USA) equipped with a cryostat. Discs cut from tested tensile specimens weighing approximately 15–20 mg were sealed in aluminum pans with perforated lids. The samples were heated from 0 °C to 250 °C at a rate of 10 °C·min^−1^, cooled at 0 °C at a rate of −10 °C·min^−1^, maintained for 5 min at this temperature, and re-heated from 0 °C to 250 °C at a rate of 10 °C·min^−1^. All scans were carried out under a constant nitrogen purge of 30 mL·min^−1^. Melting points (Mp) and crystallization temperatures (Tc) were taken as the maximum of the endothermic transition and the minimum of the exothermic transition, respectively, whereas glass transition temperatures (Tg) were taken as the midpoint of the change in heat capacity. The crystallinity of the samples was calculated by taking the enthalpy of fusion of the 100% crystalline PBT homopolymer (ΔH_0_) as 32.0 kJ·mol^−1^ (145 J·g^−1^) [31], as shown in the following equation:

$$Crystallinity\;\left(\%\right)=\frac{{∆H}_{m}}{{∆H}_{0}}\times \;100$$

where ΔH_m_ is the melting enthalpy (J·g^−1^), (or the crystallization enthalpy, ΔH_c_) measured as the area under the melting (or crystallization) peak during the DSC heating (or cooling) scan, and ΔH_0_ is the melting enthalpy for 100% crystalline PBT homopolymer (145 J·g^−1^).

Temperature and enthalpy were calibrated with the Indium standard (Mp (onset) = 156.60 °C; ΔH_m_ = 28.45 J·g^−1^).

Mechanical properties. Tensile properties were measured at ambient temperature in an Instron testing machine model 4301 (Instron, Norwood, MA, USA) equipped with a 1 kN load cell. Type 5B dumbbell test pieces (according to ISO 527-2 for tensile testing of plastics; the same test specimen as the Type 4 dumbbell according to ISO 37 for rubber) were cut from the injected plates. A crosshead speed of 5 mm·min^−1^ was used. The strain was measured using the crosshead separation and referred to 10 mm of initial length. A minimum of five samples were tested for each material. The calculation of the error in the measurements was performed using a *t*-test (two-tailed distribution with a significance level of α = 0.05).

## 3. Results and Discussion

### 3.1. PBT Characterization

Prior to irradiation, the unirradiated material was characterized for its chemical structure, thermal properties, and mechanical properties.

**Figure 1:**
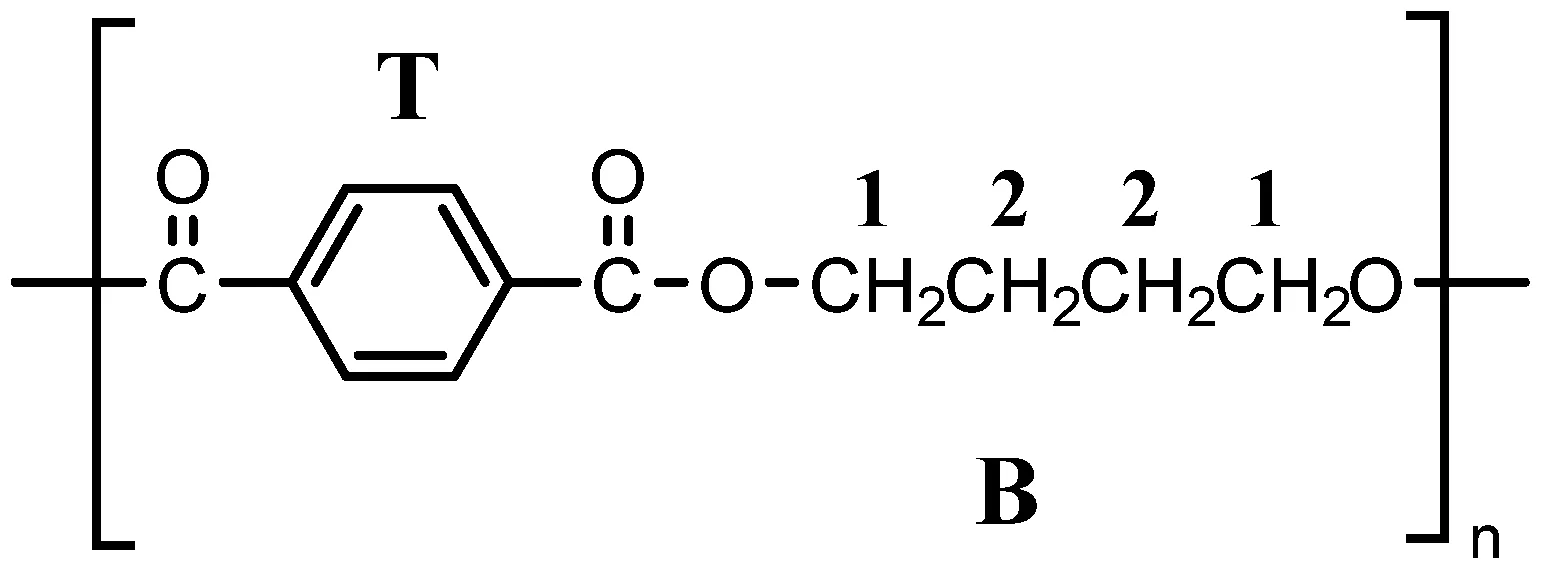
Chemical structure of PBT. T = terephthalic ring; B = butylene units.

In Figure 1, the chemical structure is presented.

PBT did not dissolve in common deuterated solvents, and some drops of HFIP were added to solubilize it. The signal of the methine proton of HFIP merged with the methylene protons of butylene (B) units linked to the ester group (protons 1 in Figure 1) and was not resolved in deuterated chloroform with HFIP. Derivatization with TFA decreased the signal for the methine proton of HFIP, and the peak for proton 1 was well defined. In Figure S2 in the Supplementary Material, the proton NMR spectrum of the unirradiated material derivatized with trifluoroacetic anhydride (TFA) is shown.

A singlet at 8.16 ppm corresponds to the four protons of the terephthalic ring (T).

The signals at 4.54 ppm and 2.09 ppm are due to butylene (B) units, methylene groups next to the ester group (protons 1 in Figure 1), and internal methylene groups (protons 2 in Figure 1). No signals for terminal groups were detected, as expected for a high molecular weight polymer.

From thermogravimetric analysis, it was found that all the material degraded without any significant amounts of residue (Figure S3 in the Supplementary Material). Decomposition started at temperatures above 300 °C; thus, at the processing temperature, the material is thermally stable.

In Figure S4 in the Supplementary Material, the calorimetric curves for PBT are shown. In the first heating (blue color in Figure S4 in the Supplementary Material), from 0 to 250 °C, at approximately 45 °C, a Tg (Tg_1_) immediately followed by a small peak is found. The peak at the end of the Tg_1_ transition is assigned to an excess enthalpy due to sub-Tg annealing of the glassy part of the polymer, as found in several amorphous polymers [32]. At approximately 160 °C, a melting endotherm starts with a small cold crystallization peak at approximately 204 °C and a maximum (Mp_1_) at approximately 225 °C. Both the excess enthalpy and the cold crystallization peaks are due to the processing conditions because of the fast cooling from the injection temperature of 265 °C to the mold temperature of 75 °C and further annealing at room temperature for a long time. On cooling from the melt (red color in Figure S4 in the Supplementary Material), a single crystallization peak (Tc) with a maximum at approximately 185 °C is found. In the second heating (green color in Figure S4 in the Supplementary Material), from 0 to 250 °C, a Tg (Tg_2_) at approximately 42 °C followed by a broad melting endotherm with two maxima at approximately 217 (Mp’_2_) and 225 °C (Mp_2_) are found.

For the crystallinity calculation, an enthalpy value for 100% crystalline PBT, experimentally calculated, of 145 J·g^−1^ was used [31]. This value is close to the theoretically calculated value (150 J·g^−1^) [33]. Calculated crystallinities for the first heating, crystallization, and second heating runs were 30%, 34%, and 37%, respectively. Thus, the overall crystallinity of PBT was relatively low, below 40%. The lower value for the first heating is due to the fast cooling during processing by injection. On cooling from the melt, the material has more time to crystallize, and the resulting crystallinity value is higher than in the first heating. On the second heating, the crystallinity value is slightly higher than the crystallinity in the cooling step, as usually found in the literature [34], because the material does not have enough time to develop full crystallization.

In Figure S5 in the Supplementary Material, the tensile stress–strain curve for PBT is shown. The properties of the injected material when the tensile specimens were cut in the direction perpendicular (as in Figure S1-right in the Supplementary Material) or parallel to the flow of the melt in the mold were tested for the 2 mm thickness. No significant differences were found in the results: 57 ± 5 MPa strength, 620 ± 50% strain, 800 ± 70 MPa Modulus; and 63 ± 4 MPa strength, 670 ± 30% strain, 840 ± 40 MPa Modulus; for the perpendicular and parallel directions, respectively. Thus, injection did not align the chains, and the injected material was isotropic. For the rest of the testing, the perpendicular direction of the melt flow was chosen for the cut of the tensile specimens.

The shape of the curve was typical for a tough thermoplastic, similar to polyethylene. After a fast increase in stress at low strain, producing a Young’s Modulus of approximately 800 MPa, the material yielded at approximately 10% strain and 45 MPa stress, with a slight decrease in stress afterwards, producing a neck (Figure S6 in the Supplementary Material, necking), followed by a pseudo-plateau from 30 to 300% strain approximately, where stress does not change significantly with strain (Figure S6 in the Supplementary Material, yielding 1,2). Above 300% strain, yielding reached the wide part of the dumbbell specimen (Figure S6 in the Supplementary Material, yielding 3), and higher force is needed to continue yielding, and therefore stress increases constantly and linearly with strain (Figure S6 in the Supplementary Material, yielding 4), until rupture at high strain (Figure S6 in the Supplementary Material, breakage), approximately 620%, reaching a high tensile strength of approximately 60 MPa. The calculated values for the mechanical properties show that, because the material is highly amorphous but with high molecular weight, it reaches high tensile strength and high strain at break.

### 3.2. Viscosimetry

PBT was not soluble in common solvents, and SEC could not be used to measure the changes in molecular weight. In order to estimate the changes in molecular weight with irradiation, inherent viscosity was measured for the non-irradiated and irradiated PBT. All samples were completely soluble in HFIP; thus, no gel was produced up to 2000 kGy irradiation, in agreement with the literature, where no gel was formed after irradiation with gamma up to 4000 kGy [20], and 10,000 kGy were needed to reach the gel point [8]. Ionizing radiation generally produces both crosslinks and chain scission [35]. However, in the case of the PBT studied here, no gel was formed, and therefore chain scission would be the dominant mechanism.

**Figure 2:**
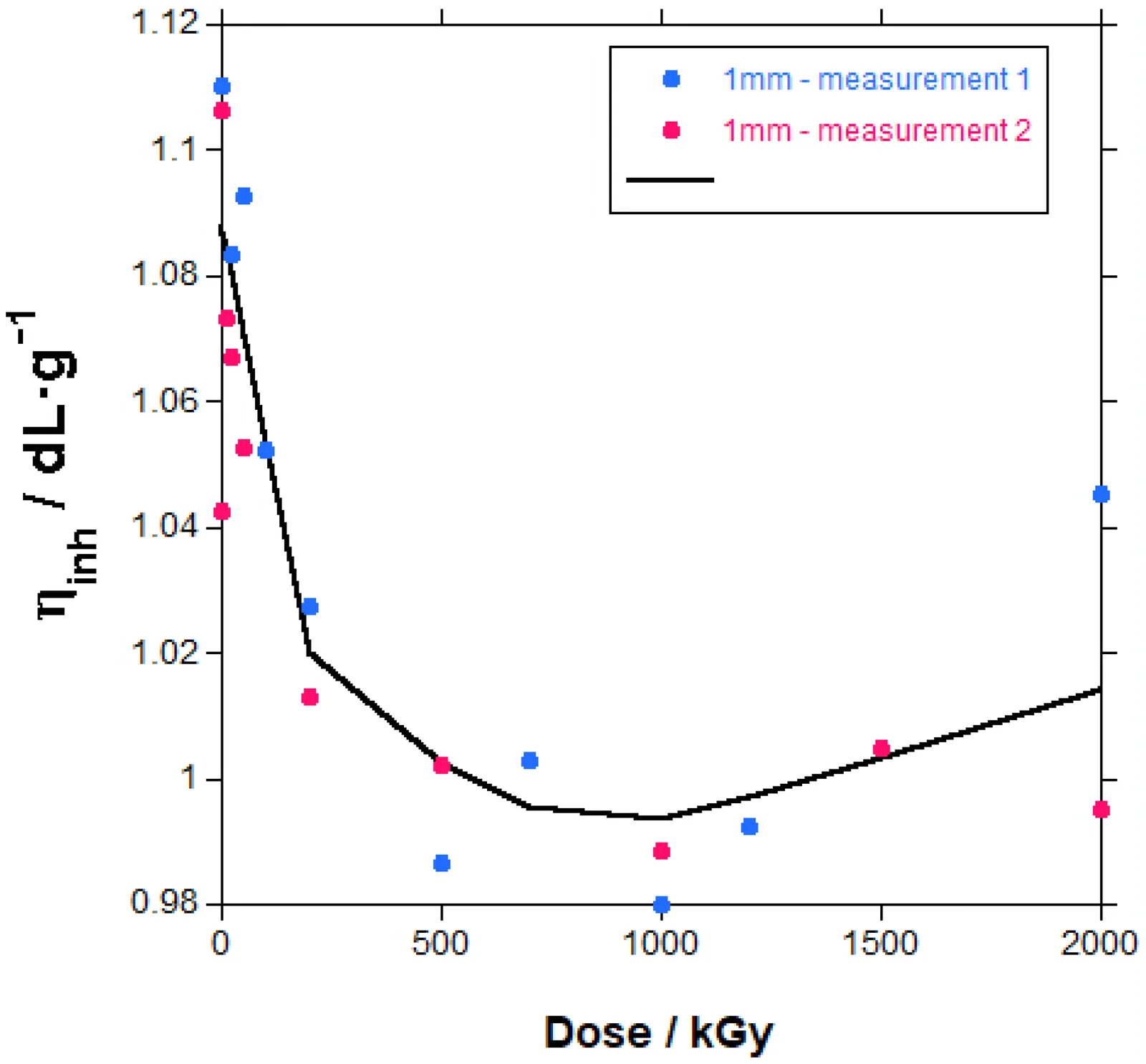
Inherent viscosity vs. irradiation dose for PBT (1 mm thickness). Black line is a guide to the eye.

This was confirmed by the data on the inherent viscosity of the samples. In Figure 2, the values of inherent viscosity for 1mm thick samples are displayed. Measurements were performed in duplicate. Despite the dispersion of the measurements, it can be seen that the molecular weight of the material decreased with the increase in dose up to approximately 1000 kGy, and then slightly increased.

Although chain scission is the dominant mechanism, crosslinking takes place, and its importance increases above 1000 kGy; the branching produced by crosslinking compensates for the decrease produced by chain scission.

Compared with the continuous decrease in the inherent viscosity for PET [36], the increase in the length of the diol (from 1,2-ethylene glycol to 1,4-butadiene diol) for PBT increased the probability of a crosslinking event (Figure S7 in the Supplementary Material). Furthermore, when terephthalate units are partially substituted by aliphatic adipate units as in poly(butylene adipate-co-terephthalate) (PBAT), the probability of crosslinking events is additionally increased and gelled material is obtained [34].

In an early study on the effect of gamma radiation in PBT, it was found that intrinsic viscosity decreased up to 2000 kGy and then increased up to 10,000 kGy, when gel was formed [8]. These results are in agreement with our results, where viscosity decreased up to 2000 kGy. The explanation for the shape of the viscosity curve vs. dose was the same as ours: initial response to gamma radiation by chain scission and increase in crosslinking ratio until predominance at the gel point at 10,000 kGy. This overall mechanism was supported by the results for poly(decamethylene terephthalate), with a much longer aliphatic chain, which increased viscosity rapidly until gelation at 2000 kGy. Thus, they concluded that the longer the aliphatic chain in the terephthalate polymer, the lower the chain scission and the higher the crosslinking, starting from PET, where chain scission was the main mechanism and crosslinking was virtually absent, leading to a continuous decrease in viscosity, as we also found [36]. Other authors also found a 50% decrease in molecular weight at a 2500 kGy dose, the single dose measured in this work [22].

In another study, the gamma-irradiated PBT molecular weight, calculated from viscosity measurements, dropped at 50 kGy, slightly increased at 300 kGy, and then decreased moderately at 845 and 1000 kGy [21]. This trend, different from our results, was also found for PET, which is known to decrease its molecular weight continuously with increasing dose, and for all the studied polyesters, aromatic and aliphatic; thus, it seems that it was a systematic error in the determination of viscosities.

Viscosity of the melt at 230 °C was evaluated for the injected irradiated pellets at different frequencies, as seen in Figure S8 in the Supplementary Material. Viscosity decreased with the increase in dose, without a significant change in the shape of the curve (almost linear in the double logarithmic scale with the same slope). When the viscosity results were plotted versus the irradiation dose at different frequencies, a continuous decrease with the increase in the irradiation dose was found, with a similar shape for all the frequencies (see Figure S9 left in the Supplementary Material for the values at 3.8 Hz as an example). If the viscosity values are plotted on a linear scale (Figure S9 right in the Supplementary Material), the decrease in viscosity is very fast until a 1000 kGy dose, and then the decrease is smaller at higher doses, similar to the trend found in Figure 2 for the inherent viscosity.

### 3.3. Proton NMR

To our knowledge, no NMR studies have been carried out so far in PBT to determine the chemical species produced by ionizing radiation.

**Figure 3:**
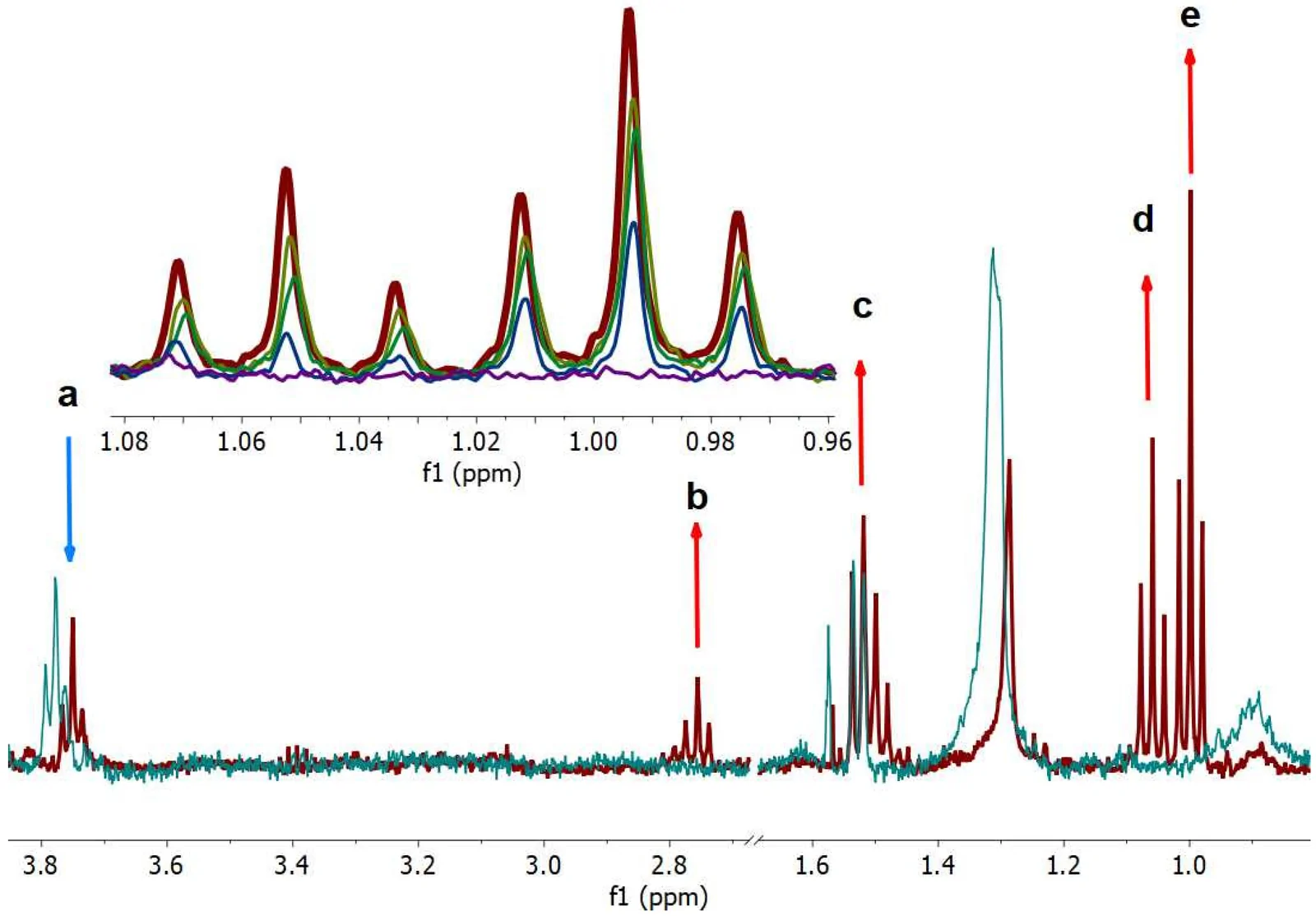
Proton NMR spectra for unirradiated PBT (blue) and PBT irradiated at 2000 kGy (red) in the aliphatic region (CDCl_3_ + HFIP, derivatized with TFA). Inset: stacked spectra of unirradiated PBT (violet) and PBT irradiated at 500 kGy (dark blue), 1000 kGy (green), 1500 kGy (gray), and 2000 kGy (red) in the range 0.96–1.08 ppm.

In Figure 3, the changes in the proton NMR spectra between the derivatized unirradiated PBT and the derivatized PBT irradiated at 2000 kGy are shown.

A small peak at approximately 3.75 ppm (a), already present on the unirradiated PBT, decreased with the increase in dose, whereas new peaks appeared and increased with the increase in dose at approximately 2.77 ppm (b, triplet), 1.52 ppm (c, multiplet), 1.06 ppm (d, triplet) and 1.00 ppm (e, triplet). None of these signals changed their displacement with derivatization significantly, and therefore, the carbon of these groups is not directly attached to a hydroxyl group that would react with TFA. The proton–proton homonuclear correlation spectrum (Figure S10 in the Supplementary Material) confirmed that signal at 3.75 ppm was correlated to a signal at approximately 1.89 submerged in the big signal for proton 2 (see Figure S2 in the Supplementary Material); signal at 2.77 ppm was correlated to a signal at approximately 2.25 submerged in the big signal for proton 2; signal at 1.52 ppm was correlated to signal at 1.00 ppm and to a signal at approximately 1.82 submerged in the big signal for proton 2; and signal at 1.06 ppm was correlated to a signal at approximately 1.87 submerged in the big signal for proton 2.

**Figure 4:**
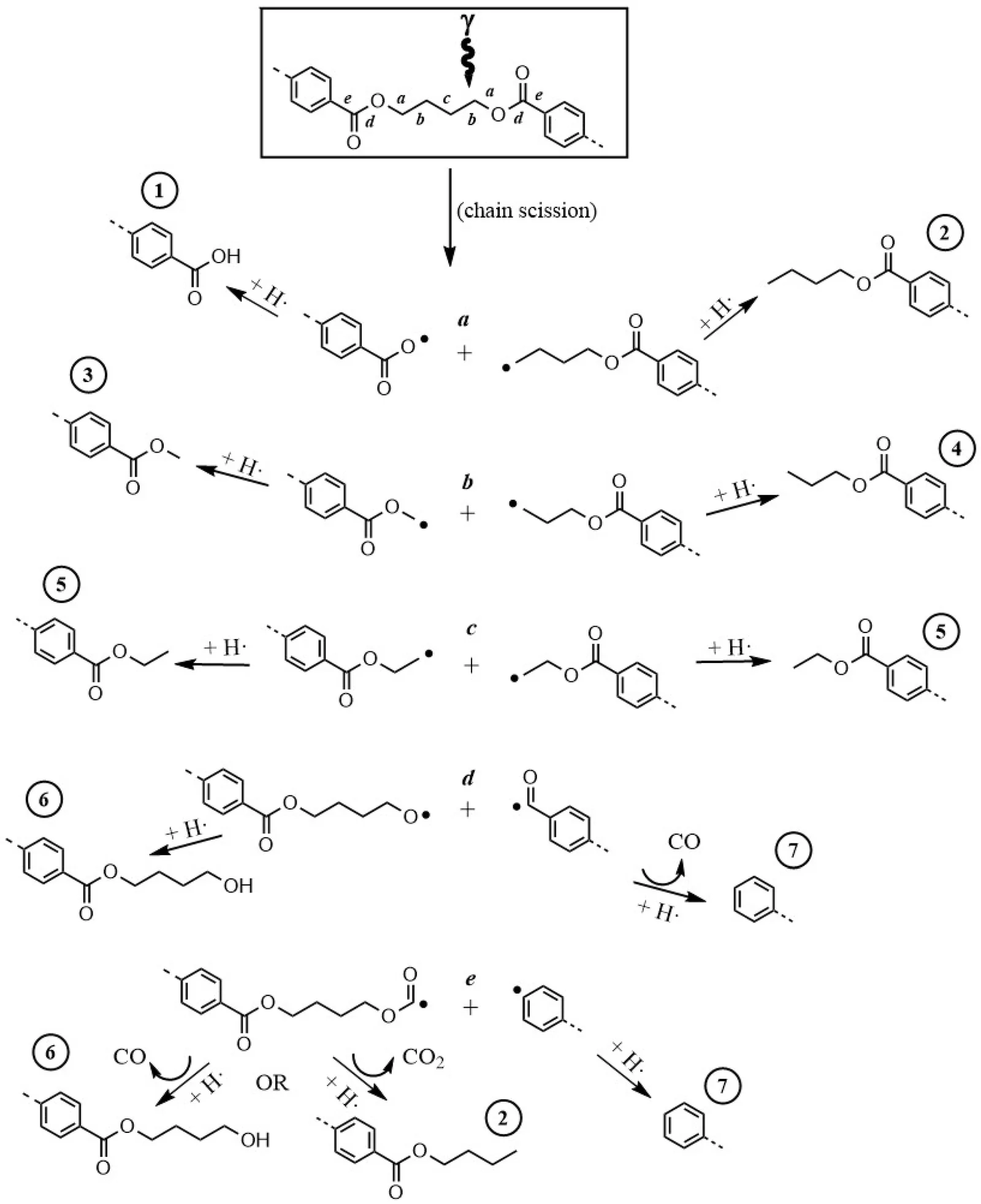
Possibilities for bond scission in PBT.

The absence of gel at 2000 kGy showed that chain scission was predominant. From the chemical structure of PBT, 5 bonds can be broken, as seen in Figure 4.

After chain scission and hydrogen abstraction, 7 possible species can be obtained.

Species ②, ④, ⑤, and ③ with butyl, propyl, ethyl, and methyl terminal groups, respectively; species ⑥ with hydroxybutyl terminal group; and species ① and ⑦ with aromatic carboxylic end groups and monosubstituted aromatic ring, respectively.

Theoretical calculations with MestReNova^®^ software (version 14.2.0-26256) showed that the methyl group for species ②, ④, ⑤, and ③ should be located at approximately 0.94, 0.99, 1.40, and 3.87 ppm, respectively. The triplet at 1.00 ppm, correlated to multiplets at 1.52 and 1.82 ppm, is in agreement with a butyl chain end, species ②, the remaining peak, expected at 4.24 ppm, is probably submerged in the big signal for protons 1 and HFIP (see Figure S2 in the Supplementary Material). The peak at 1.06 ppm, correlated to a signal at approximately 1.87 submerged in the big signal for protons 2, is in agreement with a propyl chain end, species ④, the remaining peak, expected at 4.24 ppm, probably submerged in the big signal for protons 1 and HFIP. The methyl group for species ⑤ (ethyl) is expected at 1.40 ppm, close to the peak at 1.52 ppm. The ratio for signals at 1.00 ppm/1.52 ppm should be 1.5 if only the butyl chain end contributed to these signals, and should be below 1.5 if the methyl group of ethyl chain ends was contributing to the signal at 1.52 ppm. In Figure S11 in the Supplementary Material, this ratio is plotted vs. dose. For all the graphs plotting NMR results, the data points were measured for underivatized and derivatized samples of 1 and 1.5 mm thick and are presented all together. Despite the dispersion of the data, due to the small area of the peaks and the difficulty of drawing the baseline because of the noise, especially at low doses, the ratio is around 1.5; thus, species ⑤ are not produced or are present in negligible amounts. No peaks related to species ③ and ⑥ were found, and species ⑦, if produced, should be submerged in the big aromatic peak (T in Figure S2 in the Supplementary Material). From these results, chain scission seems to take place mainly in bonds ***a*** and ***b*** (Figure 4), with predominance of rupture of bond ***a***, as seen in Figure S12 in the Supplementary Material, where the molar ratio ②:④ is approximately 2.5 at low doses and decreases with dose to approximately 2 at 2000 kGy. This ***a*** bond is the bond broken in the thermal degradation of PBT with the formation of an aromatic carboxylic chain end [37]. Besides, if bond ***b*** was broken, undetected species ③ would be expected; thus, either species ③ does not abstract a hydrogen and preferentially reacts with another radical to produce a crosslink, or the peak at 1.06 ppm comes from a different unknown chemical structure.

The peak at 3.75 ppm, with a displacement similar to the methylene group attached to a hydroxyl group, as found for PBAT, did not move to a displacement above 4.00 ppm when derivatized [34], thus, this signal is not related to species ⑥. Furthermore, it is correlated to a signal at approximately 1.89 ppm, which is consistent with the presence of dibutylenether groups by reaction of terminal hydroxybutyl groups during PBT synthesis, similar to the formation of diethylene glycol units during the synthesis of PET [38,39]. In addition, in PBAT the hydroxyl group increased with dose, whereas the peak at 3.73 ppm in PBT decreased with dose. In a previous work by our group, it was demonstrated that chain scission in poly(tetramethylene glycol) [40] was in the bonds *beta* and *gamma* to the oxygen that produced propyl and ethyl chain ends, respectively. We did not find evidence of ethyl chain-end formation; thus, in this case, propyl groups must be preferentially formed by the scission of bond *beta* to the oxygen that would add to the signal produced by scission in bond ***b***. However, at the same time, aliphatic methyl groups should also be generated by hydrogen abstraction, but the corresponding peak at approximately 3.1 ppm is absent; thus, the origin of the peak at 1.06 ppm still remained uncertain.

The peak at 2.77 ppm, correlated to a signal at approximately 2.25 ppm, could not be correlated to any species produced by chain scission.

In the aromatics region, no significant changes are found in the underivatized spectra, but in the derivatized spectra new peaks appear on the low-field side of the main aromatic single peak, as seen in Figure S13 in the Supplementary Material. These peaks were also found for PBAT [34] and are consistent with the rupture of bond ***a***, forming terephthalic anhydrides by reaction of species ① with trifluoroacetic anhydride, and similarly, the aromatic signals are partially submerged in the big terephthalate signal and cannot be integrated separately. However, it can be seen that the area of the new peaks is of the order of the area of the peak at 1.00 ppm (species ②), which comes from the same bond rupture (ratio should be 4:3 for ①: ②), and therefore supports the statement that bond ***a*** is preferentially broken and species ① and ② are produced.

The increase or the decrease in the signals vs. dose, are plotted in Figures S14–S16 in the Supplementary Material (with the same Y axis scale for easier comparison), given as % with respect to the reference signal at 2.09 ppm for butylene protons 2 in Figure S2 in the Supplementary Material, corrected for the number of protons in the signal (4 for protons 2 and peak at 3.73 ppm; 3 for peak at 1.00 and 1.06 ppm; 2 for peak at 1.52 ppm; and 1 for peak at 2.77 ppm because of the unknown origin of this peak). The molar amount of the generated species by gamma radiation is very small, less than 1% mol at a 2000 kGy dose, but as we will see later, the effect on the physical properties is huge. In Figure S14 in the Supplementary Material can be seen that peaks at 1.00 and 1.52 ppm follow the same trend and with the same molar ratio, demonstrating the correspondence of these peaks again. The trend is not linear, with a slightly lower rate when the dose increased. The peak at 1.06 ppm, assigned to terminal propyl groups, follows a linear trend with the increase in dose.

The unknown peak at 2.77 ppm, although the dispersion of the data is high (Figure S15 in the Supplementary Material), the area of the peak is significant, and the peak seems to follow a non-linear trend with a slightly lower rate when the dose increased, as for peaks at 1.00 and 1.52 ppm. The peak at 3.75 ppm, due to ether groups, decreased non linearly with the increase in dose (Figure S16 in the Supplementary Material), and at 2000 kGy, still more than 50% of the groups are intact.

**Figure 5:**
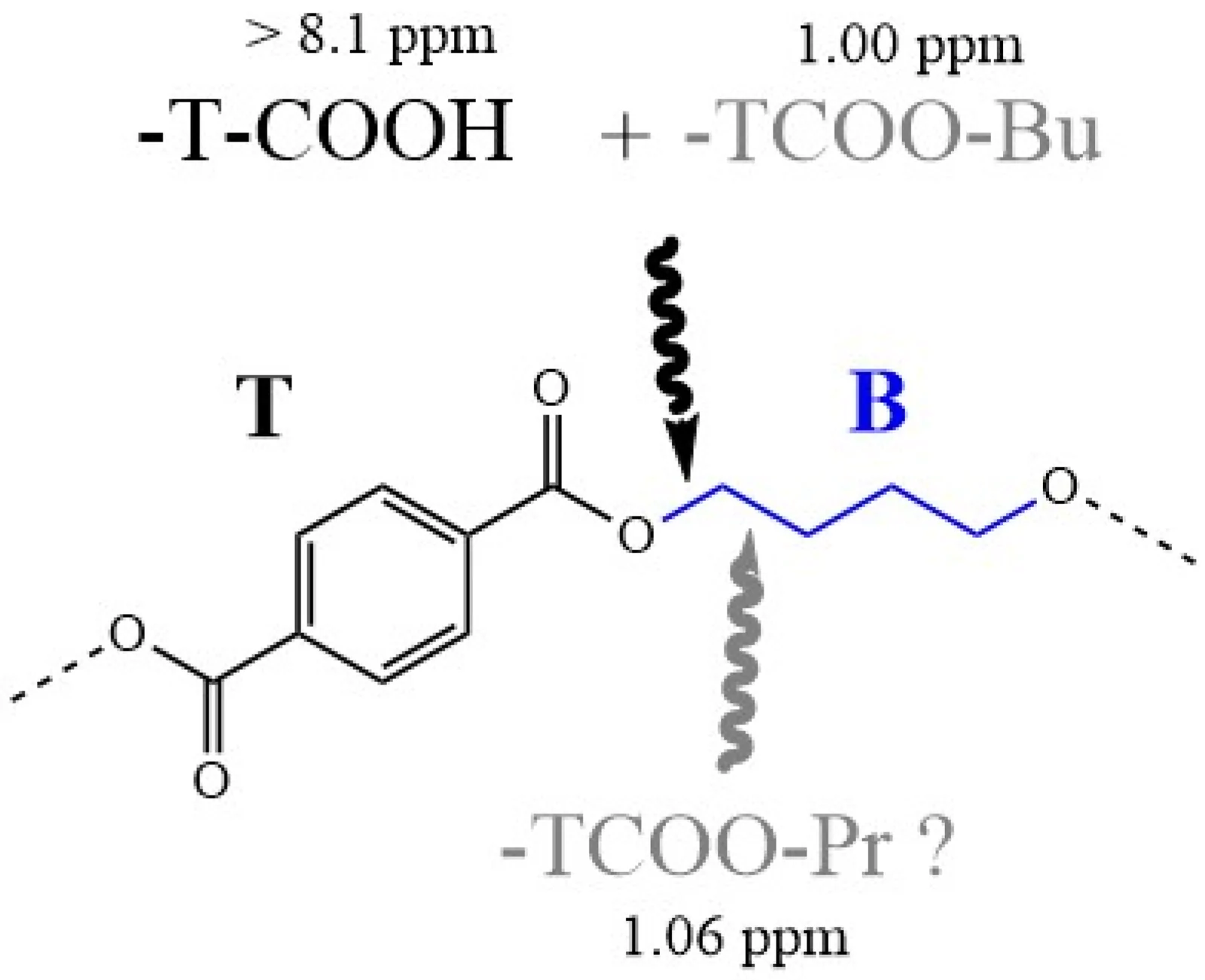
Preferential PBT bonds broken by gamma irradiation.

Summarizing, chain scission takes place preferentially in bond ***a*** and probably in bond ***b*** in the PBT chain, Figure 5, and in the bond *beta* to the oxygen in the dibutylenether groups. In the only work found in the literature using NMR, the ratio of T protons to 2 protons, see Figure 1, after irradiation at 2500 kGy, was found to decrease by approximately 14%, confirming the preferential rupture of aliphatic butylene chains [22]. Although we have found the same trend, Figure S17 in the Supplementary Material, and despite the dispersion of the data, the decrease we found was much smaller, around a 2% decrease at 2000 kGy, taking the values of the linear fit. The discrepancy could be due to the difference in the delay time between pulses. We know that, for polymers, a relatively long delay has to be used for the protons to completely relax. If the delay time is not long enough, part of the protons will not be relaxed, and in the following pulse, the integral will be smaller than it should be. In our experience, a 5 s time delay, which we use in our NMR experiments, is enough for polymers, and maybe the authors used a shorter delay time and the butylene protons did not completely relax, and therefore their integral was smaller than it should be.

In order to find the origin of the peak at 2.77 ppm, the theoretical displacements of the protons of the possible species formed by crosslinking of PBT chains, Figure S18 in the Supplementary Material, and crosslinking of the radicals from butylene chain ends and PBT chains, Figure S19 in the Supplementary Material, were calculated with MestReNova^®^ software. Viscosity measurements showed that crosslinking became significant at doses above 500 kGy, but no significant changes were found in the proton NMR spectra in the region where methyl groups appear; thus, species ⑧, ⑨ and ⑩ apparently are not produced. Crosslinking of species A and B, and coupling of these radicals with species ②, Figure S19 in the Supplementary Material, do not generate terminal methyl groups, and only the crosslinkings A + B and B + B would produce methine groups with displacements at 2.16–2.34. Thus, the radical species B (*beta* carbon to the oxygen) seems the most probable species to be generated in the PBT chain leading to crosslinking. In addition, coupling of radicals A and ⑧ is the only combination that could generate propyl groups that could explain the peak at 1.06 ppm.

In the literature, when the gases evolved during irradiation of PBT with gamma radiation up to 4000 kGy were analyzed, the main component was hydrogen (more than 80%); thus, hydrogen abstraction was taking place at the aliphatic chains [20], leading to the formation of the species described in Figure 4 after chain scission. The most abundant gases after hydrogen were carbon monoxide and carbon dioxide (9% of the total each). From Figure 4, CO_2_ should come from rupture of bond ***e***, and CO from rupture of bonds ***d*** and ***e***, but species ⑥ could not be found; thus, CO must come from a different mechanism than rupture of these bonds. These authors concluded that chain scission took place in the ester groups and, based on the negligible amount of released hydrocarbons (methane, ethane, propane, propene, butane), that there was no rupture in the aliphatic chains. Based in NMR results, we agree with the first statement (rupture of bond ***a***), but not in the second statement because the generation of hydrocarbon species would need the rupture of two bonds in the same aliphatic butyl chain, which is very unlikely, and because the presence of peak at 1.06 ppm assigned to propyl groups could be due to the rupture of bond ***b*** in the aliphatic chain.

### 3.4. Thermal Properties

PBT samples for thermal properties evaluation were irradiated at 0 (unirradiated), 10, 20, 30, 50, 100, 200, 500, 700, 1000, 1200, 1500, and 2000 kGy. Differential Scanning Calorimetry (DSC) was used to examine how gamma radiation affected the thermal behavior of PBT. In the first heating step, from 0 °C to 250 °C at 10 °C min^−1^, the glass transition (Tg_1_), melting point (Mp_1_), and the crystallinity of the irradiated material (χ_1_) were recorded. In the cooling step, from 250 °C to 0 °C at a rate of −10 °C min^−1^, the crystallization temperature (Tc) and the crystallinity (χ_c_) were measured. In the second heating step, from 0 °C to 250 °C at 10 °C min^−1^, the effect of irradiation on the glass transition temperature (Tg_2_), melting temperature (Mp_2_), and crystallinity (χ_2_), was evaluated. All results are listed in Tables S1–S3 in the Supplementary Material.

We have demonstrated in several irradiated polymers [34,36,40,41,42,43,44,45] that thermal properties show a very high data dispersion in general, and therefore, several repetitions have to be carried out in order to determine real tendencies in the properties. For this work, we did not carry out several runs for each sample. Instead, we recorded, for each dose, a run for each thickness. We did not find any significant influence in the results due to the difference in thickness that would be systematic when individually plotted *versus* dose. The dispersion of the data with dose was much higher than any effect that could be due to thickness. In all the graphs, results for the three different specimen thicknesses are plotted together, and the results were adjusted to a linear fit to show the average trend except when noted.

In Figures S20–S22 in the Supplementary Material, the curves for PBT unirradiated and irradiated at 2000 kGy in the first heating cycle, the cooling cycle, and the second heating cycle, respectively, are compared. As can be seen, the shape of the curves did not change upon irradiation. Results for Tg, Tm, Tc, and crystallinity (χ) are shown in Figures S23–S25 in the Supplementary Material.

Tg_1_ and Tg_2_, Figure S23 in the Supplementary Material, showed high data dispersion, with a not significant decreasing trend for Tg_1_ and a not significant increasing trend for Tg_2_, and a narrow range of variation of less than 5 °C, with a mean value across the irradiation range of 43 °C for both sets of Tg data; thus, they remain virtually unchanged with the increase in dose. Mp_1_ and Mp_2_, Figure S24 in the Supplementary Material, showed lower data dispersion than Tg, with a decreasing linear trend when irradiation dose increased, which is considered significant. Adjusted values for Mp_1_ and Mp_2_ at each dose are very close, approximately a 0.5 °C difference; thus, it can be considered that Mp did not change between the heating runs. The total decrease in temperature is very low, approximately 3 °C at a 2000 kGy dose. The secondary melting peak at a lower temperature than the main melting peak, found in the second heating run (Mp_2_), follows the same trend with the same total decrease as the main peak, Figure S26 in the Supplementary Material. Crystallinity (χ_1_, χ_c_, χ_2_), with relatively low data dispersion despite the broadness of the endotherm, ranging from 140–160 °C to around 240 °C, which makes it difficult to draw the baseline, increased fairly linearly with the increase in dose, Figure S25 in the Supplementary Material, a trend considered significant with a maximum increase in the adjusted crystallinity of 4% at 2000 kGy.

**Figure 6:**
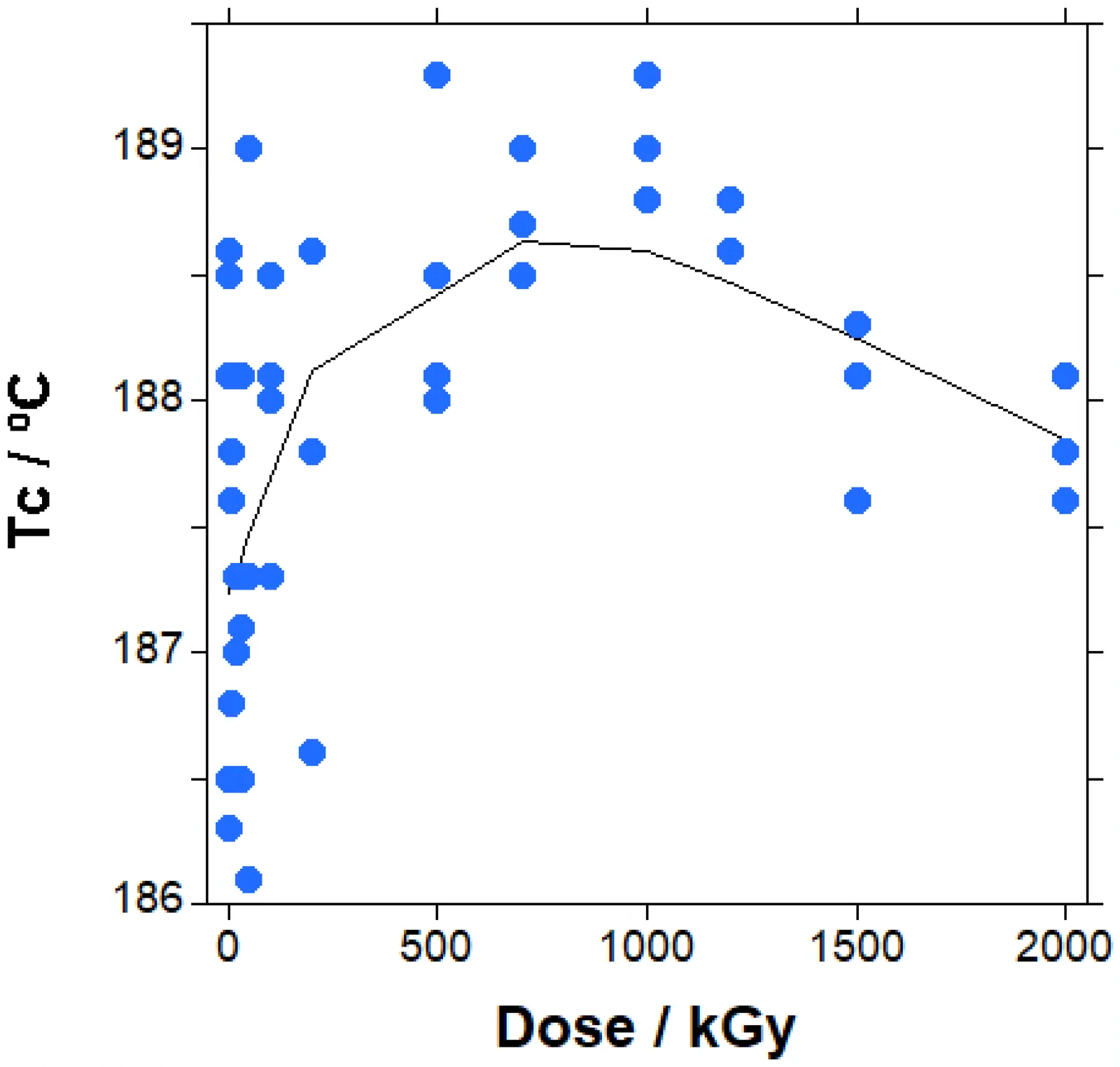
Crystallization peak (Tc) for PBT irradiated at different doses. The drawn line is just a guide to the eye.

Tc behaved very differently from the other calorimetric parameters. As can be seen in Figure 6, with an increase at low doses up to approximately 1000 kGy and then slightly decreased until 2000 kGy. Although the dispersion of the data is high (very high at low doses), it follows the exact opposite trend of the data for viscosimetry, and for this reason the trend is considered significant.

All the results for thermal properties can be explained by the effect of chain scission and crosslinking.

The predominant effect of radiation in PBT is chain scission, which affects the amorphous phase more strongly than the crystalline phase in semicrystalline polymers, and that crystals boundaries are particularly affected. The resulting shorter chains, with higher mobility, can rearrange better, resulting in the slight increase in crystallinity (χ_1_, χ_c_, χ_2_), but because the crystals are smaller by the chain scission on the boundaries or because the increase in chain ends makes it more difficult to obtain larger crystals from the melt, Mp decreased in the first heating run (Mp_1_) and in the second heating run (Mp_2_), respectively. For Tg (Tg_1_ and Tg_2_), higher crystallinity and crosslinking would reduce chain mobility and increase Tg, whereas a higher amount of chain ends would increase chain mobility and decrease Tg, but changes in all these parameters are relatively low, and they seem to compensate, without a significant change in Tg with irradiation dose.

In the case of Tc, branching due to crosslinking affected the crystallization from the melt. At low doses, up to 1000 kGy, chain scission was predominant and shorter chains facilitated crystallization at higher temperatures, and Tc increased, whereas at higher doses than 1000 kGy, crosslinking increased relative to chain scission, making crystallization more difficult and Tc decreased. The same behavior, more evident, was found for PBAT [34], where only PBT sequences crystallized, with the change in the trend *circa* the same dose.

Our results agree with literature results for the melting points (Mp_1_, Mp_2_, Mp’_2_), with a decrease of approximately 2–3 °C when PBT was irradiated to 1000–1100 kGy [8,21,26]. For crystallization temperature, Tc, only a single datum at 1100 kGy showed an increase [26], in agreement with our results. And for crystallinity, a previous study reported a decrease at 300 kGy from the 43% initial crystallinity, no change at 845 kGy, and an increase at 1000 kGy without reaching the initial crystallinity [21]. This behavior does not fit our findings: a slight and continuous increase in crystallinity up to 2000 kGy.

Compared with PET [36] and PBAT [34], melting points decreased continuously with increasing dose, as also found in all our studies on irradiation of other polymers [40,41,42,44,45]; thus, it seems that this effect is general when a semicrystalline polymer is irradiated with ionizing radiation. For Tc, it always increased for PET, and never reached an upturn as for PBT, because the increase in the length of the aliphatic chain in PBT increased the possibility of crosslinking, that is not significant for PET; and for PBAT, for which only PBT sequences can crystallize, the behavior of Tc was similar to PBT but more evident for the increase in crosslinking for PBAT with respect to PBT, as already mentioned. Tg value was statistically constant for PET, PBT, and PBAT, showing that the effect of chain scission on crystallinity and the effect of crosslinking in the amorphous phase were not significant enough to change Tg significantly. And crystallinity increased for PET and PBT, more influenced by chain scission than by crosslinking, but for PBAT χ_1_ and χ_c_, increased because chain scission was the predominant effect, but χ_2_ slightly decreased, showing a significant effect of crosslinking (higher than for PBT) when PBAT was crystallized from the melt.

### 3.5. Mechanical Properties

Mechanical properties for PBT dumbbell samples cut from injected plates of different thicknesses, after exposure to gamma irradiation at doses 10, 20, 30, 50, 100, 200, 500, 700, 1000, 1200, 1500, and 2000, were evaluated. Young’s modulus, tensile strength (stress), and elongation at break (strain) were the three main mechanical characteristics that were measured. Among all possible mechanical tests, tensile tests are the most sensitive to the molecular modifications induced by irradiation [46]. All results are listed in Tables S4–S7 in the Supplementary Material.

The shape of the stress-strain curve did not change with respect to the unirradiated material, and the main effect was the decrease in strain leading to a decrease in the ultimate strength, as can be seen in Figure S27 in the Supplementary Material.

**Figure 7:**
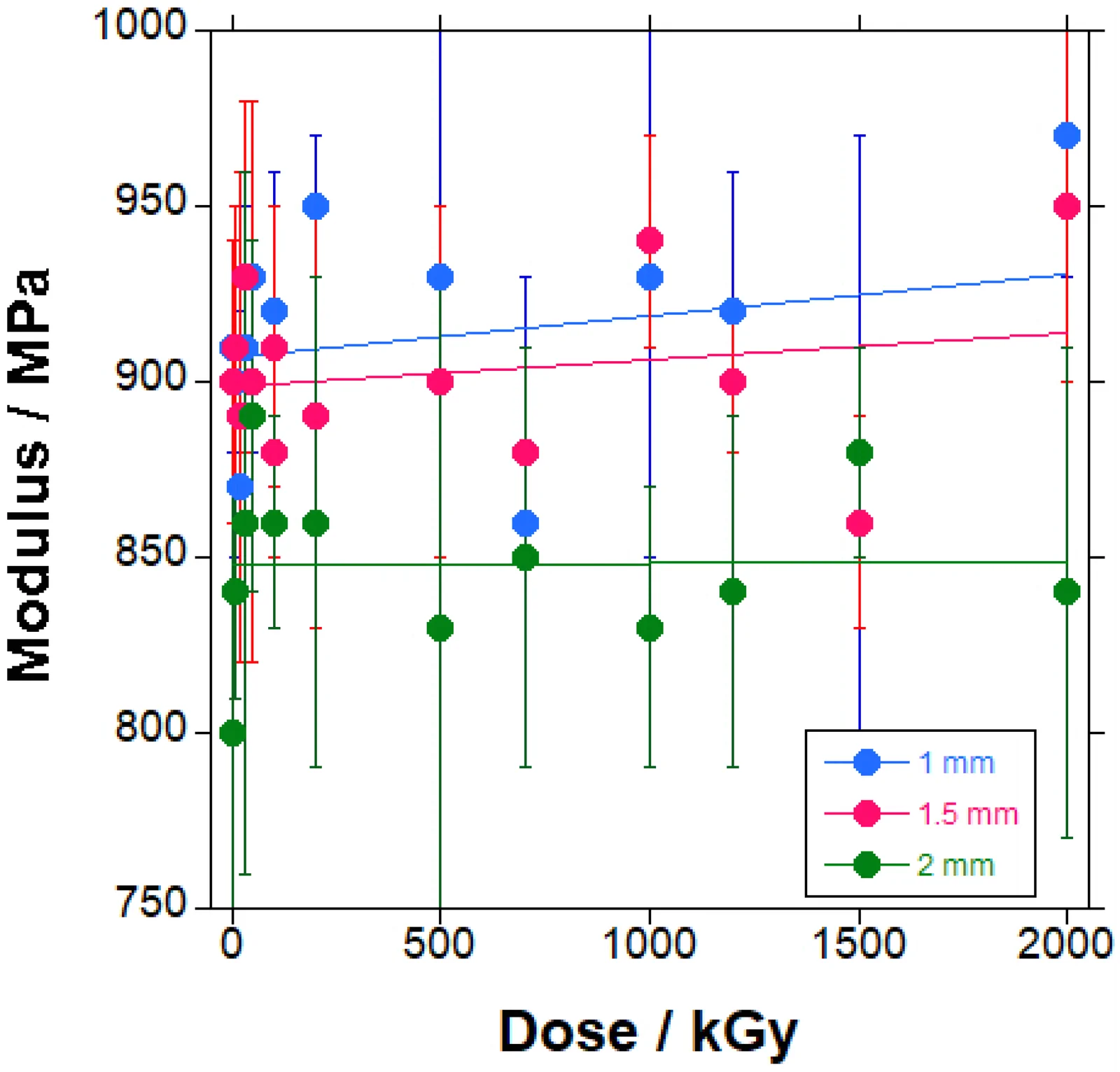
Young’s Modulus vs. dose for PBT irradiated at different doses. Linear fittings are a guide to the eye.

**Figure 8:**
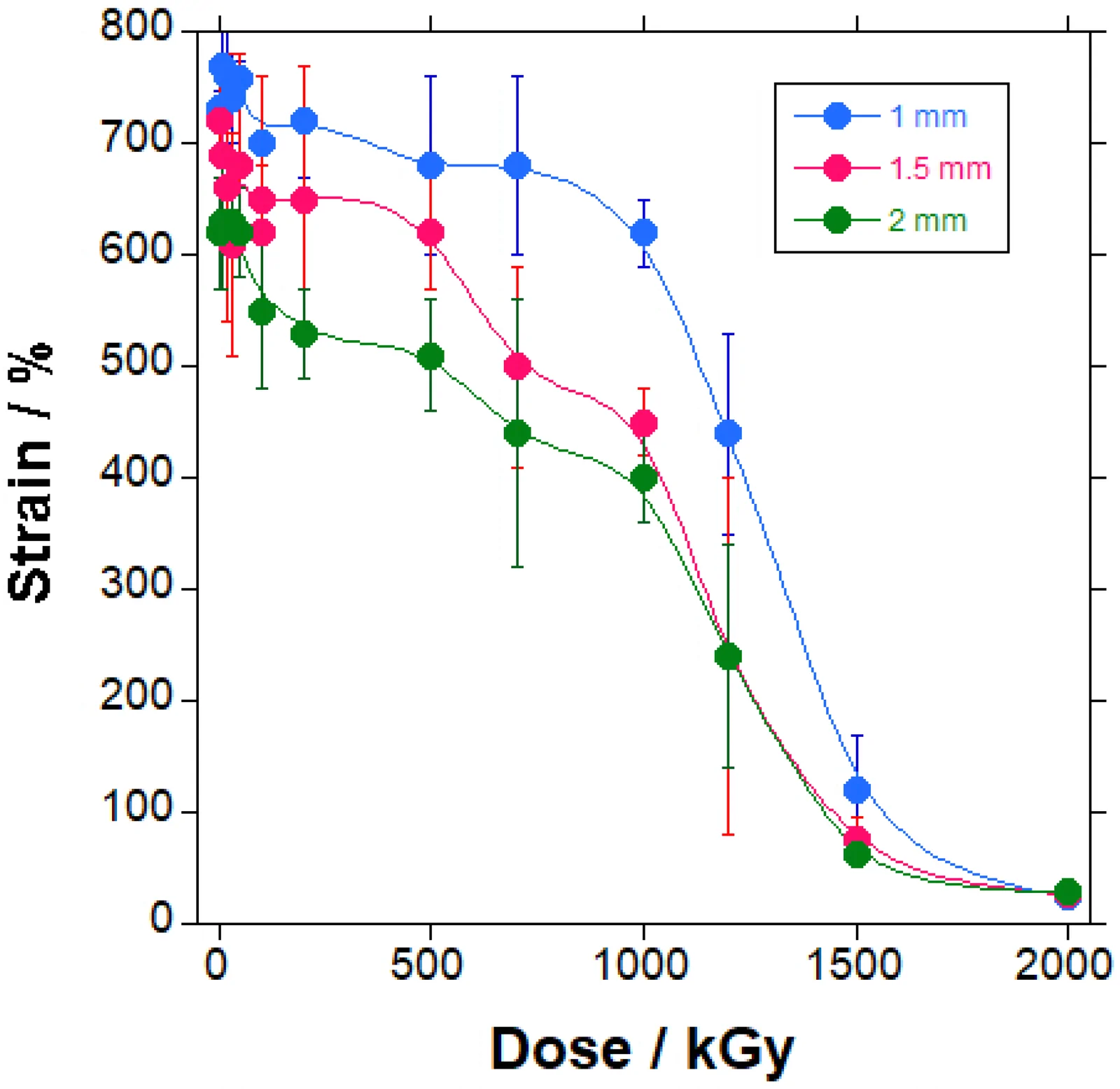
Elongation at break vs. dose for PBT irradiated at different doses.

**Figure 9:**
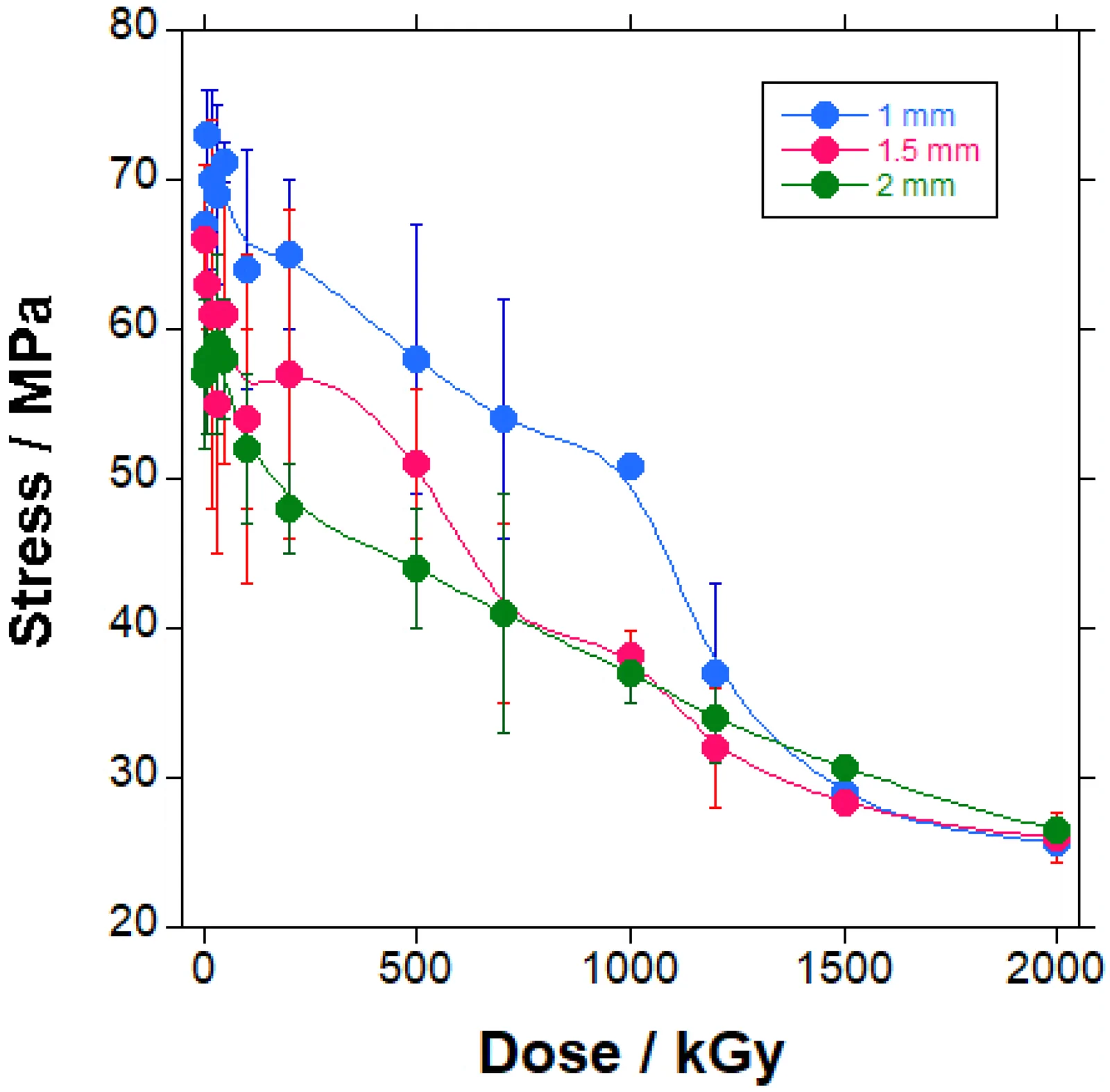
Tensile strength vs. dose for PBT irradiated at different doses.

In Figure 7, Figure 8 and Figure 9, the variation in Young’s Modulus, ultimate strain, and tensile strength with the increase in dose are presented, respectively. It has to be emphasized that crystallinity, which is a key parameter for mechanical properties, has to be taken from the first heating step because it is the crystallinity of the tested material. Despite the data dispersion, especially in the Moduli data, it is clear from the graphs that there is an effect of the specimen thickness on the mechanical properties, with higher ultimate strain and stress at a lower thickness at the same dose. It is expected that the thinner the specimen, the higher the ultimate strain that can be reached, and because the shape of the curve did not change with dose, a higher tensile strength will be attained. For the Young’s Modulus, differences are much lower than the error; thus, differences are not significant.

Young’s Modulus, shown in Figure 7, did not change significantly with dose. Crystallinity increased only slightly with the increase in dose (Figure S25 in the Supplementary Material), and this increase was not enough to have a substantial effect on Young’s Modulus.

Elongation at break, shown in Figure 8, is a measure of a material’s ability to undergo plastic deformation before failure. It suffered a slight decline up to approximately 1000 kGy and then a sharp decrease until 1500 kGy, when the material became fragile, and strain at break was very low, with rupture taking place just after yielding. This change was coincident with the change in viscosity, where branching by crosslinking became significant. The combination of crosslinking, which reduces the slippage of the chains when stretched, reducing the strain, and chain scission, which takes place preferentially in the amorphous phase and the boundaries of the crystals, reducing the connections between the crystals and amorphous phase, reduced the extensibility of the chains, anticipating the rupture at lower strains. Stress at break, Figure 9, decreased with the decrease in strain, as expected.

No data for the change of PBT mechanical properties with irradiation was found except for a mention that elongation at break decreased by 50% when irradiated at 1000 kGy in inert atmosphere and at 700 kGy in oxygen atmosphere, This decrease is in agreement with our results (Figure 8) but the decrease was lower in our case, difference that could be due to the differences in molecular weight of PBT or to the differences in the testing (size of the specimen, rate, …), not given by the authors [27].

Because, in the irradiated specimens, the effect of irradiation focuses on the amorphous phase and the boundaries of the crystals, it was hypothesized that, if the irradiated material was processed, the chain ends and the branching points could be distributed throughout the material, and its effect could be diffused, and mechanical properties improved. For this reason, irradiated PBT pellets were injected at different doses, and mechanical properties were measured for 1 mm thick specimens, which were more sensitive to changes in properties. In Figure S28 in the Supplementary Material, the injected plates for the irradiated pellets are shown. A color change that increased with the increase in dose, and that could be sensed in the irradiated dumbbell specimens, was evident in these plates, as already described by other authors [20].

If the mechanical properties of the irradiated and injected pellets are compared with the irradiated dumbbell specimens of 1 mm thickness (Figures S29–S31 in the Supplementary Material), it seems that processing of the irradiated PBT has an effect on the mechanical properties. No sharp decrease in strain at break is found above 1000 kGy dose (Figure S30 in the Supplementary Material) and the decrease in strain with the increase in dose is steady, as for the stress at break (Figure S31 in the Supplementary Material; datum for 2000 kGy dose has a very high error because only 2 specimens could be cut from the plate due to its fragility, and was not included in the fitting). From low doses to 1200 kGy, where both sets of data crossed, processing of irradiated PBT decreased the properties, whereas at 1500 kGy properties are slightly better for injected irradiated PBT. At 2000 kGy in both cases, the material is already too fragile. Thus, it seems that chain ends and branching, after processing of the irradiated PBT, are concentrated in the amorphous region, and properties were decreased further at low-intermediate doses. Also, in this dose range, crystallinity seems to be reduced, and Young’s Modulus decreased (Figure S29 in the Supplementary Material).

In Figures S32 and S33 in the Supplementary Material, the results for tensile strength and elongation at break for PBT, in terms of the retention of the property in comparison with other irradiated materials studied in the Elastomers’ group in Spain, are presented, respectively. PBT shows a very good resistance to ionizing radiation, only second to PET [36], and better than PBAT [34], where part of the aromatic terephthalic rings have been substituted by aliphatic adipate units, and than other aliphatic polymers such as PU [42], PA-6 [41], aliphatic polyesters (PCL, PBS, and PBSA) [44,45] and aliphatic polyether (pTHF) [40]. Thus, the higher the aromatic content, the better the resistance to ionizing radiation (PET vs. PBT vs. PBAT).

## 4. Conclusions

The irradiation of poly(butylene terephthalate) (PBT) with gamma rays up to 2000 kGy modified the properties of the material without gel formation.

Molecular weight changed as measured by viscosimetry and melt rheology with a decrease up to approximately 1000 kGy due to the predominance of chain scission, followed by an approximately constant value due to the increase in the ratio of crosslinking, leading to substantial branching.

The analysis by proton NMR proved that chain scission in PBT took place mainly in the ester bond, producing terminal carboxylic and butyl end groups, with a significant amount of propyl end groups consistent with the scission of the aliphatic bond *beta* to the oxygen, with both species increasing with the increase in dose. Dibutylene ether groups, coming from the synthesis of PBT, were broken by gamma radiation, and their content decreased with the increase in dose.

Thermal properties were differently affected depending on the predominant effect. Crosslinking took place mainly in the amorphous phase, whereas chain scission took place in the amorphous phase and at the boundaries of the crystals, leaving smaller crystals and shorter chains with higher mobility that crystallized more easily, leading to a significant melting point decrease and a slight increase in the amount of crystallinity. The effect of chain scission predominated in the crystallization temperature until a dose of approximately 1000 kGy, after which it increased; at higher doses, branching produced by crosslinking became significant, and crystallization temperature decreased.

Irradiation strongly influenced the mechanical properties except for the Young’s Modulus, that remained virtually unaltered without a significant influence by the slight increase in crystallinity. Stress at break and strain at break were continuously reduced with the increase in dose until a fragile material was obtained due to the combined effect of the crosslinking in the amorphous phase and the chain scission on the boundaries of the crystals.

The results in this work provide an understanding of the mechanism of crosslinking and chain scission in PBT, and its relationship with the changes in molecular weight and thermal properties and with the continuous degradation of the mechanical properties until embrittlement, providing for the first time a full set of data to predict the behavior of this polymer when subjected to high-energy radiation.

## Data Availability

The raw data supporting the conclusions of this article will be made available by the authors on request.

## Supplementary Materials

The following supporting information can be downloaded at: https://www.mdpi.com/article/10.3390/polym18172123/s1. Figure S1: Injected sample from which test specimens were cut; Figure S2: Proton NMR spectrum of PBT with some drops of HFIP and derivatized with TFA; Figure S3: TGA curve for unirradiated PBT. Inset: stacked spectra of unirradiated PBT (violet), and PBT irradiated at 500 kGy (dark blue), 1000 kGy (green), 1500 kGy (grey) and 2000 kGy (red) in the range 0.96–1.08 ppm; Figure S4: DSC curves for unirradiated PBT (1 mm thickness); Figure S5: Stress-strain curve for unirradiated PBT (2 mm thickness; cut in the direction perpendicular to the melt flow); Figure S6: Stages on the tensile test (from top to bottom, from left to right): necking; yielding 1 (constant stress); yielding 2 (constant stress); yielding 3, reaching the wide part of the specimen (increase in stress); yielding 4, increase in stress; breakage; Figure S7: Percentage of inherent viscosity respect to unirradiated material vs. irradiation dose for PBT (1 mm thickness) and PET; Figure S8: Melt viscosity at 230 °C of injected irradiated pellets vs. frequency; Figure S9: Melt viscosity at 230 °C of injected irradiated pellets measured at a frequency of 3.8 Hz vs. irradiation dose. Left: viscosity in logarithmic scale; right: viscosity in linear scale; Figure S10: Proton–proton homonuclear correlation spectrum for PBT irradiated at 2000 kGy in the aliphatics region; Figure S11: 1.00 ppm/1.52 ppm signals ratio vs. dose; Figure S12: 1.00 ppm/1.06 ppm signals ratio vs. dose; Figure S13: Proton NMR spectra for derivatized unirradiated PBT (blue curve) and PBT irradiated at 2000 kGy (red curve), magnified in the aromatics region (left) and the aliphatics region (right) with the same intensity scale; Figure S14: Mol % of the peaks at 1.00 ppm (blue colour), 1.52 ppm (black colour) and 1.06 ppm (red colour) respect to the reference peak for protons 2 at 2.09 ppm. Adjusted lines for peaks 1.00 and 1.52 (quadratic) are just a guide to the eye; Figure S15: Mol % of the peak at 2.77 ppm with respect to the reference peak for protons 2 at 2.09 ppm. Adjusted line (quadratic) is just a guide to the eye; Figure S16: Mol % of the peak at 3.75 ppm with respect to the reference peak for protons 2 at 2.09 ppm. Adjusted line (quadratic) is just a guide to the eye; Figure S17: Ratio or aromatic protons (T) to butylene (B) protons; Figure S18: Possible species formed by crosslinking of radicals produced in PBT main chain; Figure S19: Possible species formed by crosslinking of radicals produced in PBT main chain with radicals produced in the butyl chain ends; Table S1: Calorimetric properties for irradiated PBT—1 mm thick specimens; Table S2: Calorimetric properties for irradiated PBT—1.5 mm thick specimens; Table S3: Calorimetric properties for irradiated PBT—2 mm thick specimens; Figure S20: DSC curves in the first heating for unirradiated 1 mm thick PBT (blue color) and for 1 mm thick PBT irradiated at 2000 kGy (red color); Figure S21: DSC curves in the cooling for unirradiated 1 mm thick PBT (blue color) and for 1 mm thick PBT irradiated at 2000 kGy (red color); Figure S22: DSC curves in the second heating for unirradiated 1 mm thick PBT (blue color) and for 1 mm thick PBT irradiated at 2000 kGy (red color); Figure S23: Tg (Tg_1_ for the first heating run and Tg_2_ for the second heating run) vs. dose for irradiated PBT at different doses. Linear fitting of the data is just a guide to the eye; Figure S24: Main melting peak (Mp_1_ for the first heating run and Mp_2_ for the second heating run for irradiated PBT vs. dose; Figure S25: Main melting peak (Mp_1_ for the first heating run and Mp_2_ for the second heating run for irradiated PBT vs. dose; Figure S26: Secondary melting peak (Mp’_2_) for the second heating run for irradiated PBT vs. dose; Table S4: Mechanical properties for irradiated PBT—1 mm thick specimens; Table S5: Mechanical properties for irradiated PBT—1.5 mm thick specimens; Table S6: Mechanical properties for irradiated PBT—2 mm thick specimens; Table S7: Mechanical properties for irradiated PBT pellets subsequently injected– 1 mm thick specimens; Figure S27: Stress-strain curves for PBT irradiated at different doses; Figure S28: Injected plates from PBT pellets irradiated at different doses; Figure S29: Young’s Modulus vs. dose for irradiated PBT dumbbell specimens 1 mm thick (1 mm) and injected irradiated PBT pellets (Pellet 1 mm); Figure S30: Elongation at break vs. dose for irradiated PBT dumbbell specimens 1 mm thick (1 mm) and injected irradiated PBT pellets (Pellet 1 mm); Figure S31: Tensile strength vs. dose for irradiated PBT dumbbell specimens 1 mm thick (1 mm) and injected irradiated PBT pellets (Pellet 1 mm); Figure S32: Retention of tensile strength vs. dose for PBT (black filled circles), PBAT (dark grey filled diamonds), PBS (purple circles), PBSA (grey circles), PCL 50K (red circles), PCL 80K (blue circles), PA-6 (green squares), PET (dark grey filled squares), aliphatic polyurethane (magenta diamonds) and poly(tetramethylene oxide) (orange squares); and Figure S33: Retention of tensile strain vs. dose for PBT (black filled circles), PBAT (dark grey filled diamonds), PBS (purple circles), PBSA (grey circles), PCL 50K (red circles), PCL 80K (blue circles), PA-6 (green squares), PET (dark grey filled squares), aliphatic polyurethane (magenta diamonds) and poly(tetramethylene oxide) (orange squares).
