# Supplementary material for: Effect of Gamma Radiation on the Chemical Structure and Physical Properties of Poly(butylene terephthalate) (PBT)
Source: Polymers (Basel). 2026 Aug 31;18(17):2123. doi: 10.3390/polym18172123 (PMC13568323; doi:10.3390/polym18172123)
Supplement: Supplementary file 1 [file polymers-18-02123-s001.zip › polymers-4515378-supplementary.pdf]

Supporting Information

Effect of Gamma Radiation on the Chemical Structure and Physical Properties of Poly(butylene terephthalate) (PBT)

Daniel Marcos Rios [1], Guillermina Burillo [2], Rodrigo Navarro [1] and Ángel Marcos-Fernández [1,*]

[1] Institute of Polymer Science and Technology (ICTP-CSIC), Juan de la Cierva, 3, 28006 Madrid, Spain;
d.marcos@ictp.csic.es (D.M.-R.); rnavarro@ictp.csic.es (R.N.)
[2] Instituto de Ciencias Nucleares, Universidad Nacional ⍰utónoma de México, Circuito Exterior s/n, Ciudad Universitaria, Delegación Coyoacán, Mexico City C.P. 04510, Mexico; burillo@nucleares.unam.mx
* Correspondence: author: amarcos@ictp.csic.es

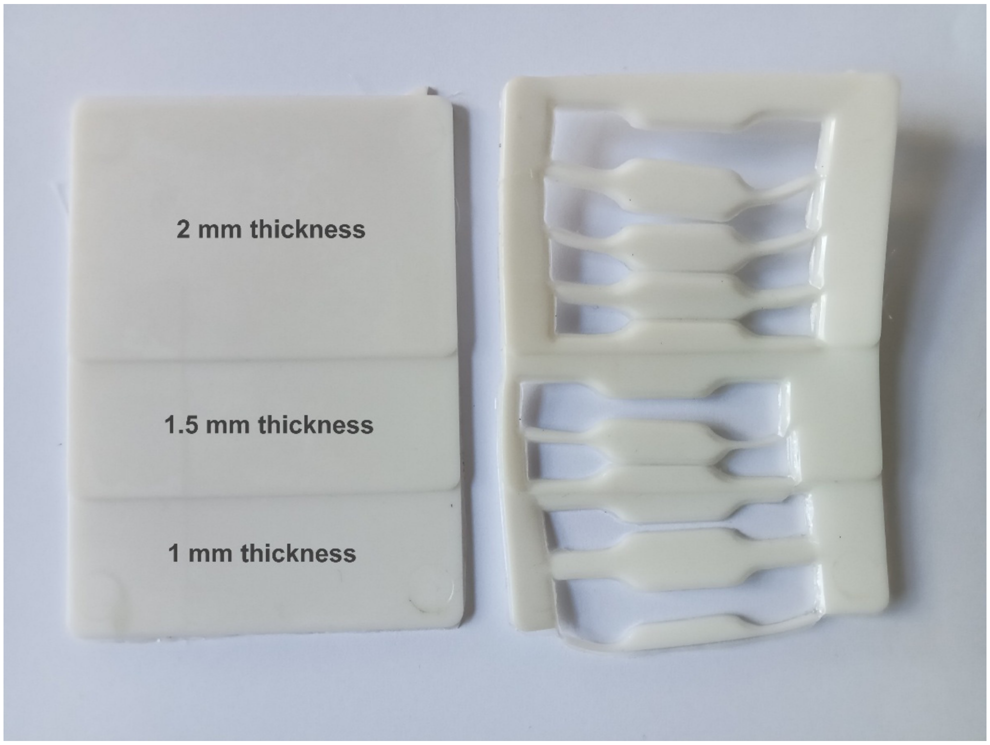

Figure S1: Injected sample from which test specimens were cut

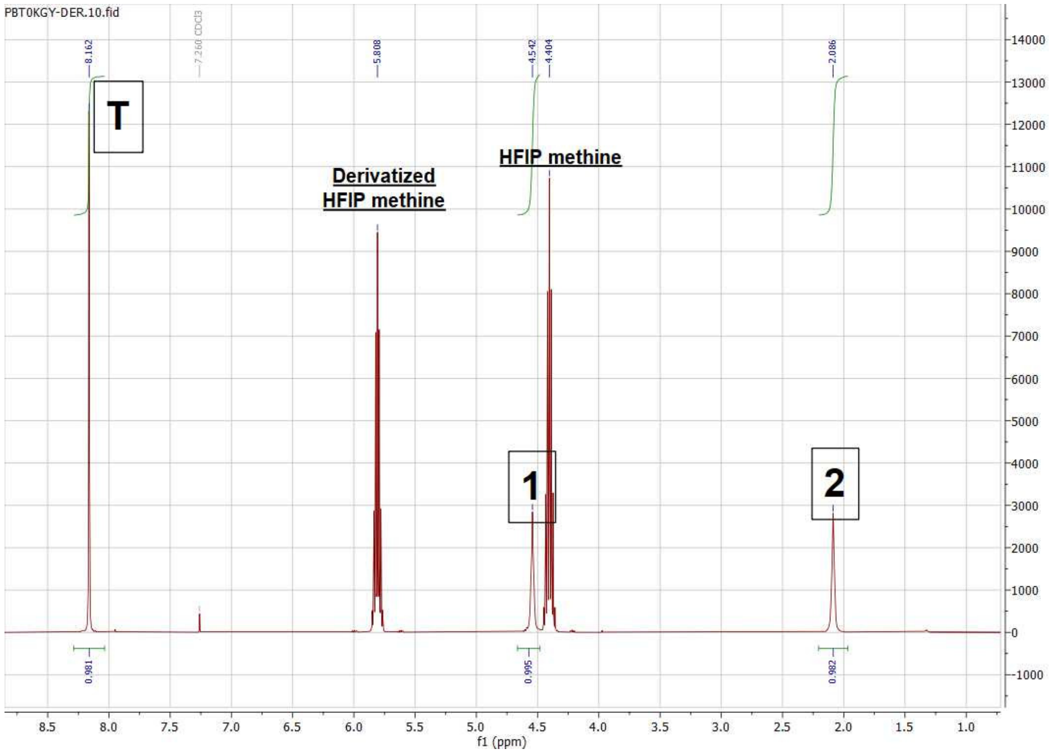

Figure S2. Proton NMR spectrum of PBT with some drops of HFIP and derivatized with TFA

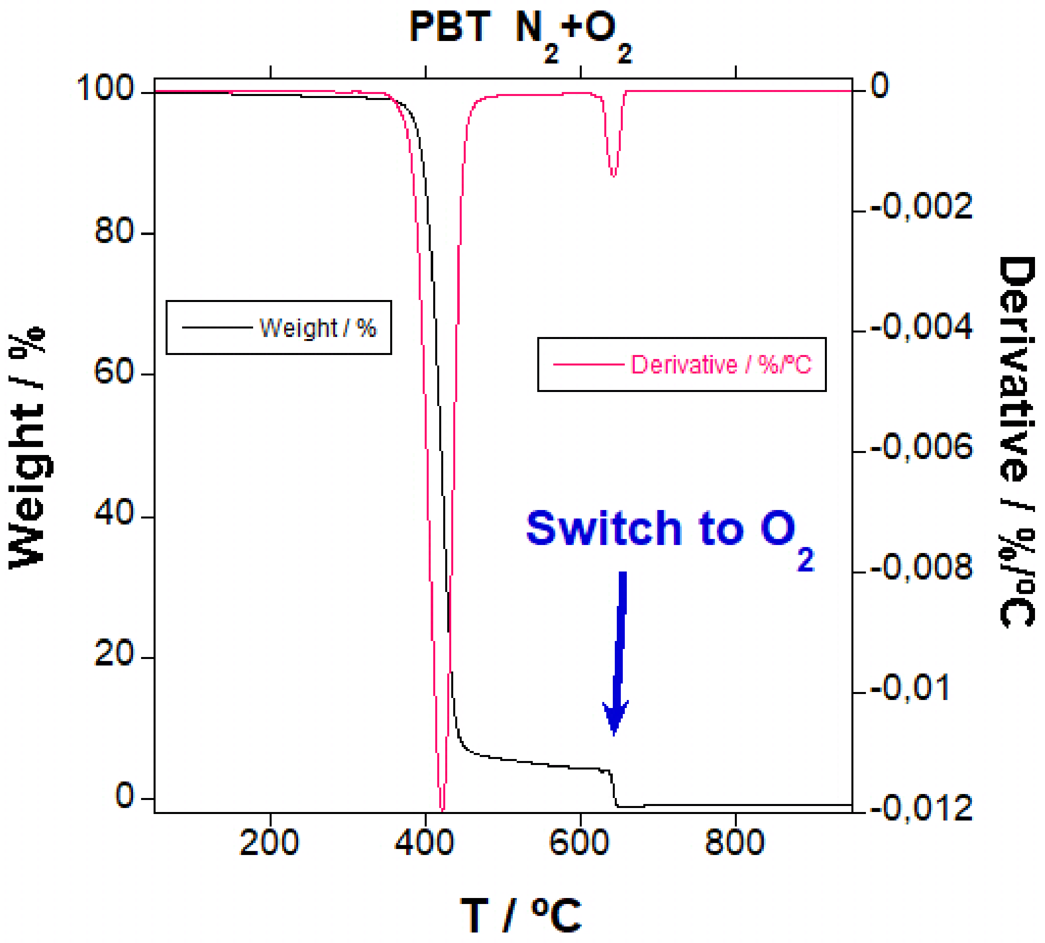

Figure S3. TGA curve for unirradiated PBT

Thermogravimetry was carried out in a Mettler Toledo instrument (Mettler Toledo, Schwerzenbach, Switzerland) model TG�/SDT�851e. � disc cut from a tested tensile specimen weighing 10.86 mg, was heated from 50 °C to 625 °C at a rate of 10 °C·min^{-1}, in a nitrogen atmosphere, and at 625 °C, the atmosphere was changed to oxygen and heating continued at a rate of −10 °C·min^{-1} until 950 °C.

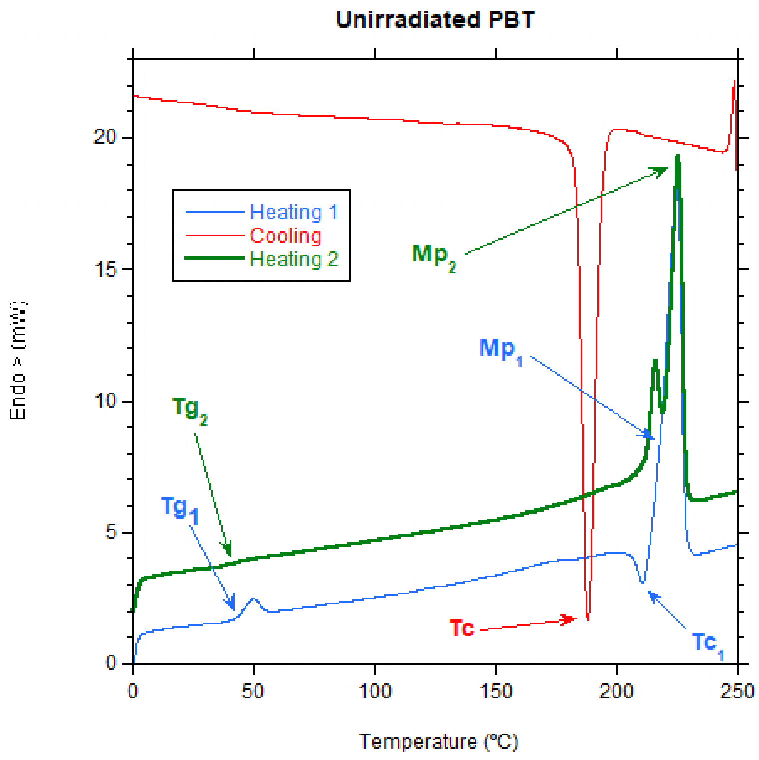

Figure S4. DSC curves for unirradiated PBT (1mm thickness)

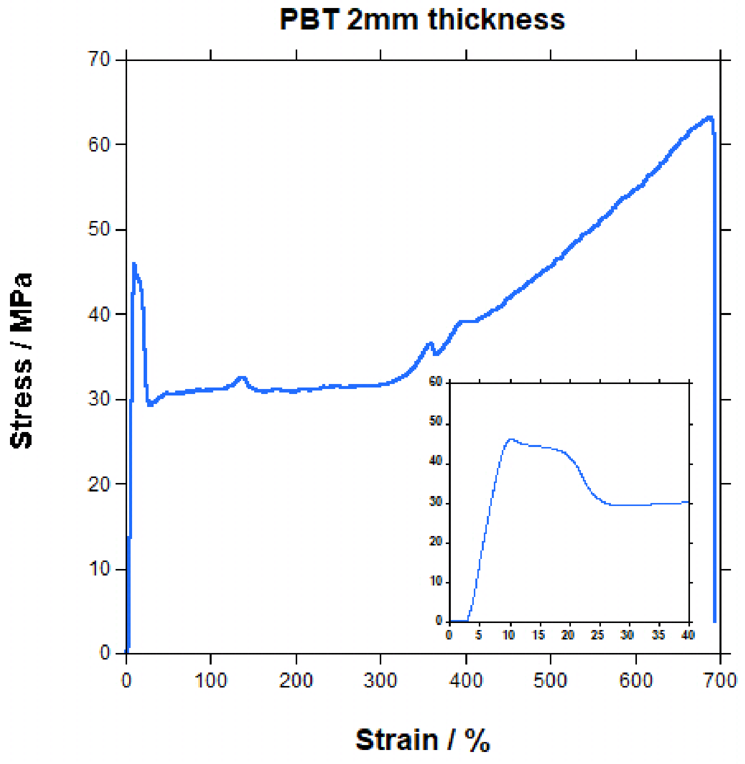

Figure S5. Stress-strain curve for unirradiated PBT (2mm thickness; cut in the direction perpendicular to the melt flow)

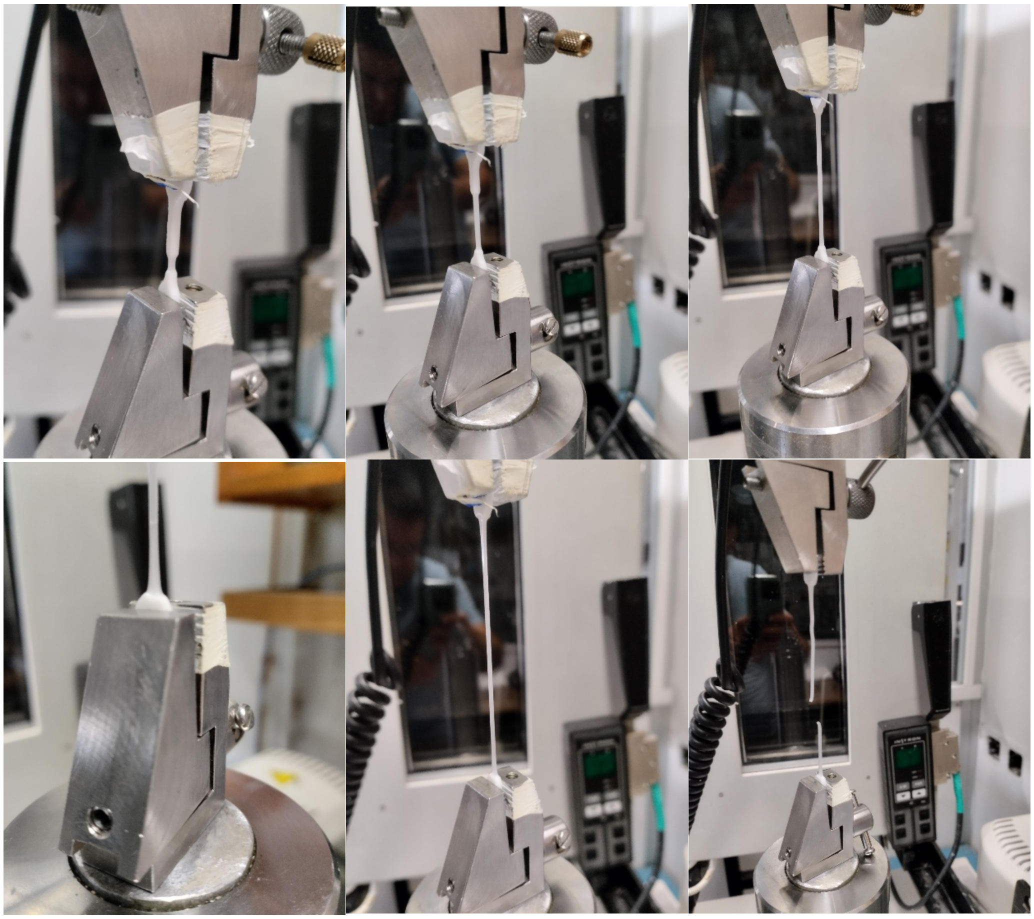

Figure S6. Stages on the tensile test (from top to bottom, from left to right): necking; yielding 1 (constant stress); yielding 2 (constant stress); yielding 3, reaching the wide part of the specimen (increase in stress);); yielding 4, increase in stress; breakage

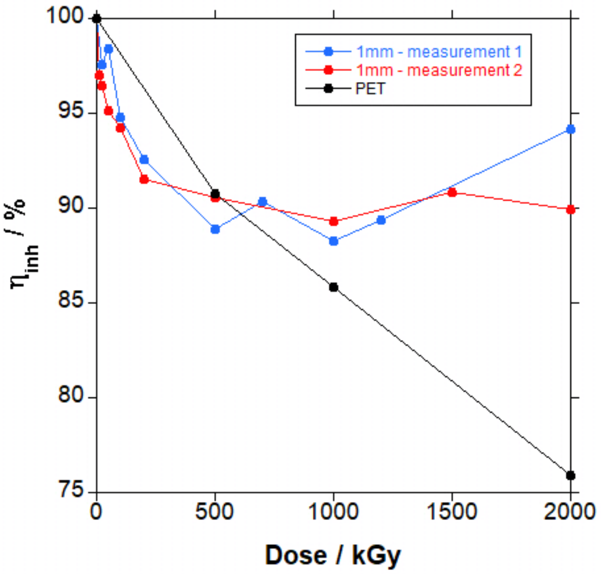

Figure S7. Percentage of inherent viscosity respect to unirradiated material vs irradiation dose for PBT (1mm thickness) and PET

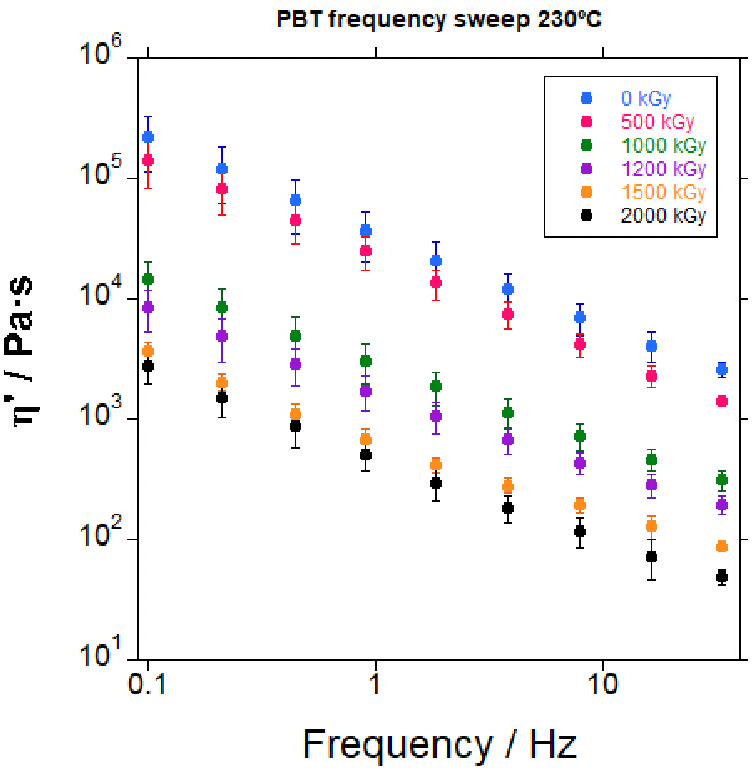

Figure S8. Melt viscosity at 230 °C of PBT injected irradiated pellets vs frequency

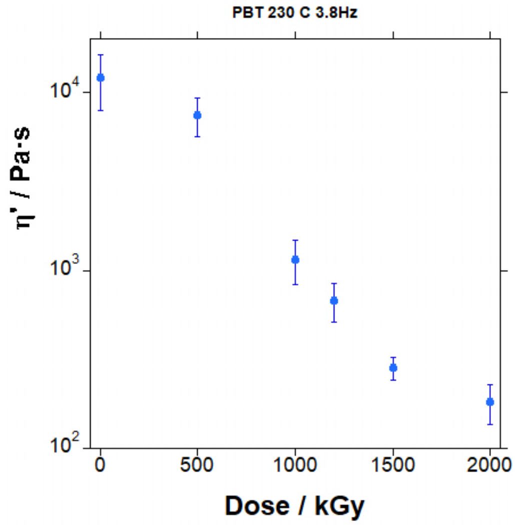

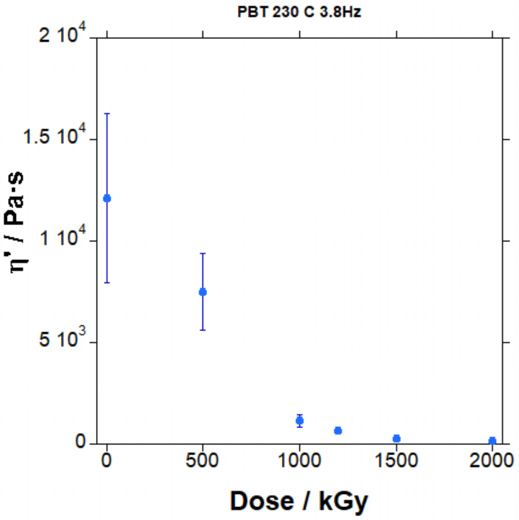

Figure S9. Melt viscosity at 230 °C of the PBT injected irradiated pellets measured at a frequency of 3.8 Hz vs irradiation dose. Left: viscosity in logarithmic scale; right: viscosity in linear scale

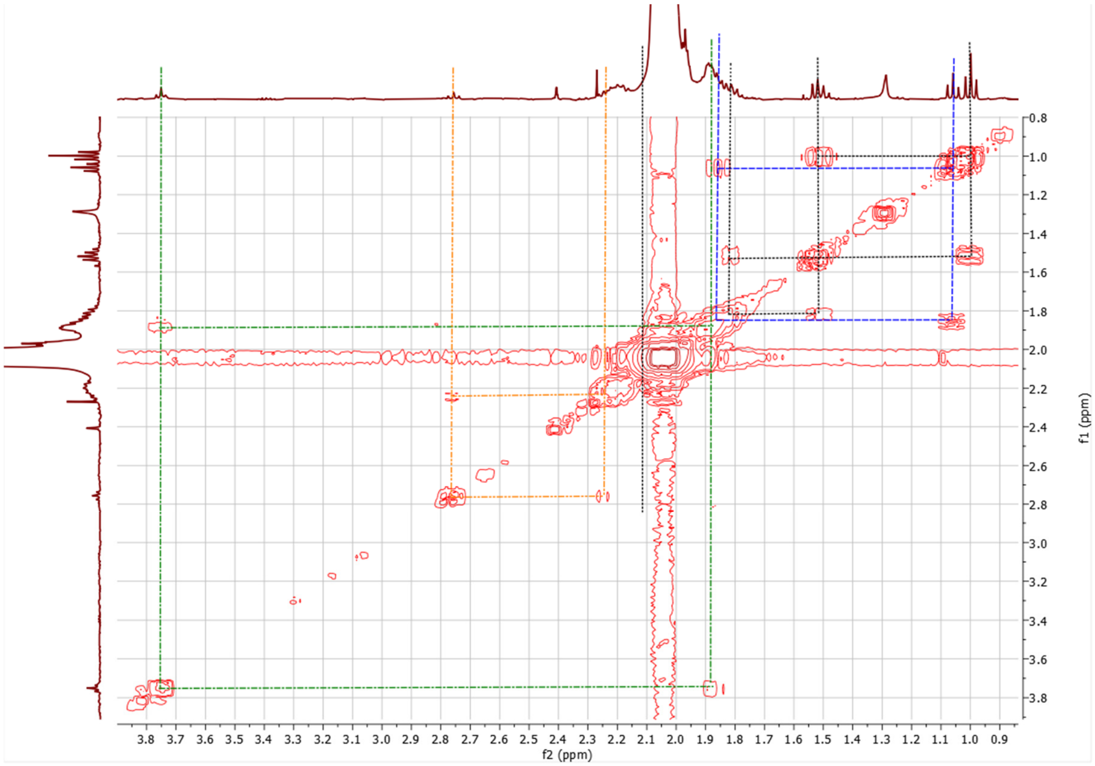

Figure S10. Proton–proton homonuclear correlation spectrum for PBT irradiated at 2000 kGy in the aliphatics region.

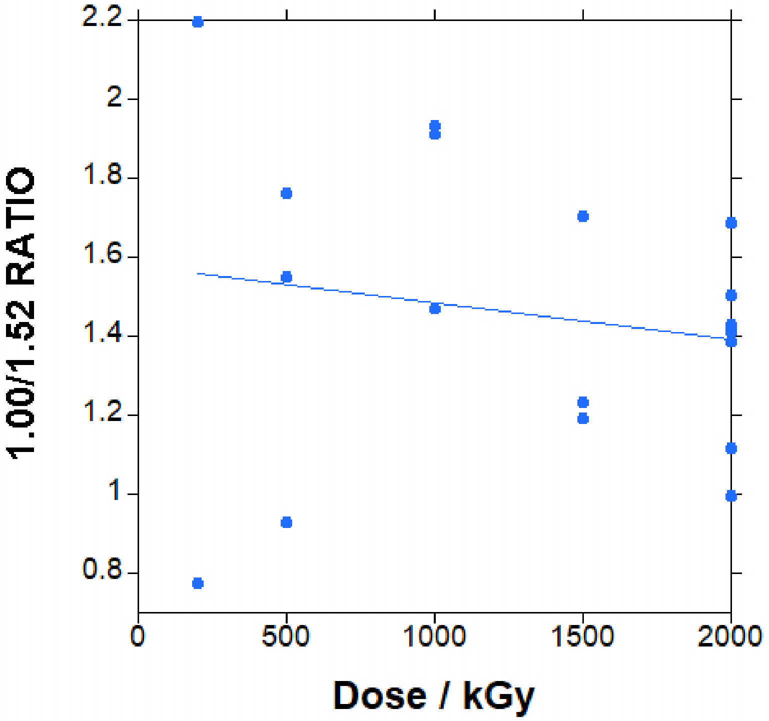

Figure S11. 1.00 ppm/1.52 ppm signals ratio vs dose

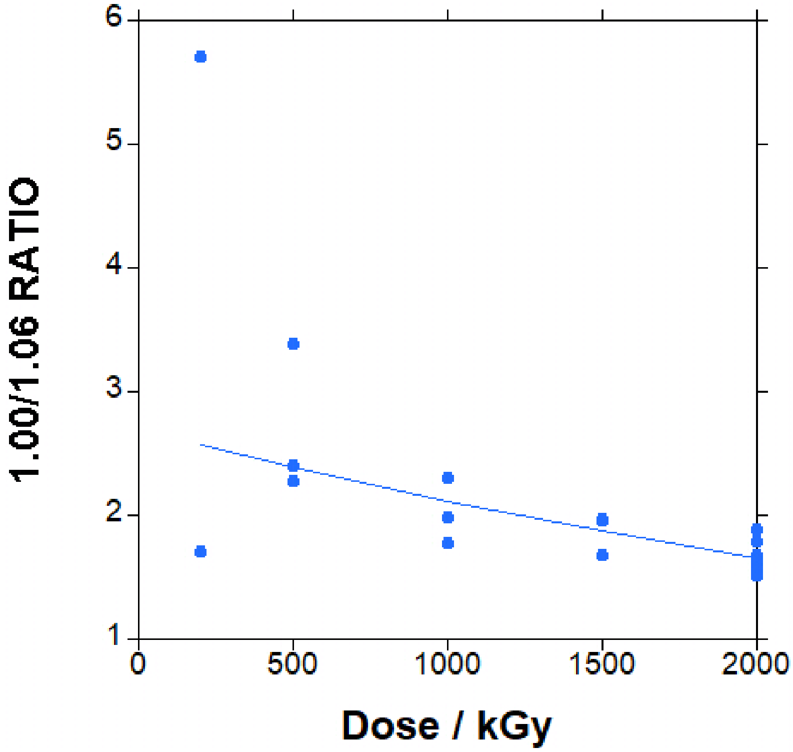

Figure S12. 1.00 ppm/1.06 ppm signals ratio vs dose

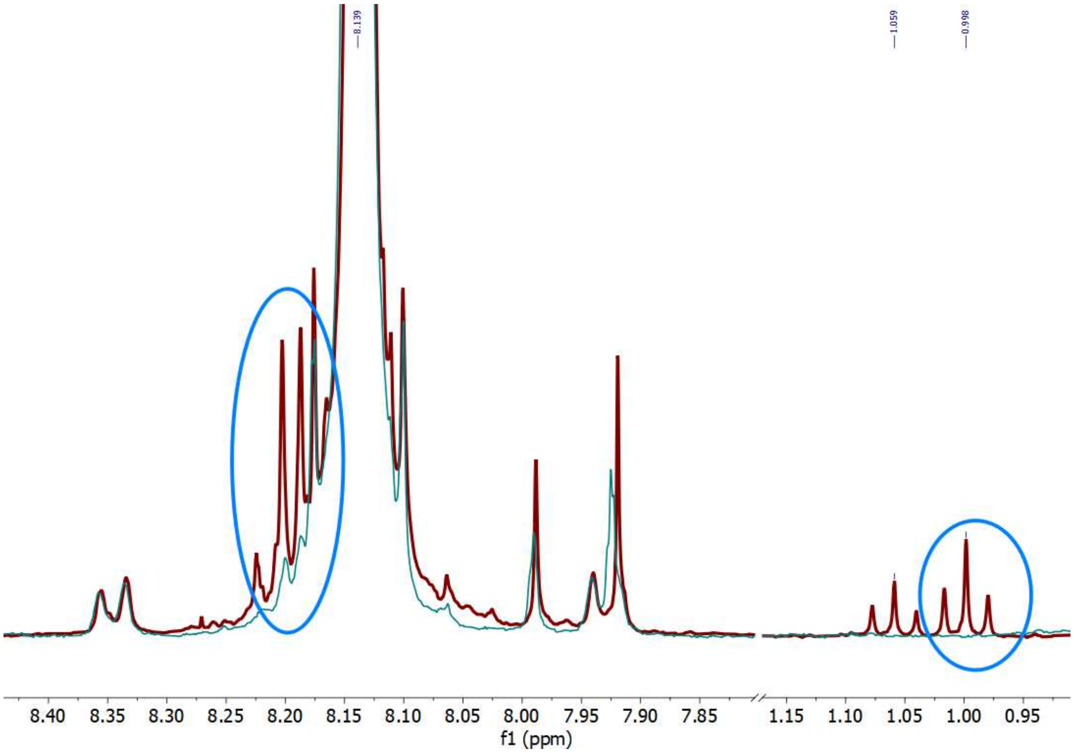

Figure S13. Proton NMR spectra for derivatized unirradiated PBT (blue curve) and PBT irradiated at 2000 kGy (red curve), magnified in the aromatics region (left) and the aliphatics region (right) with the same intensity scale

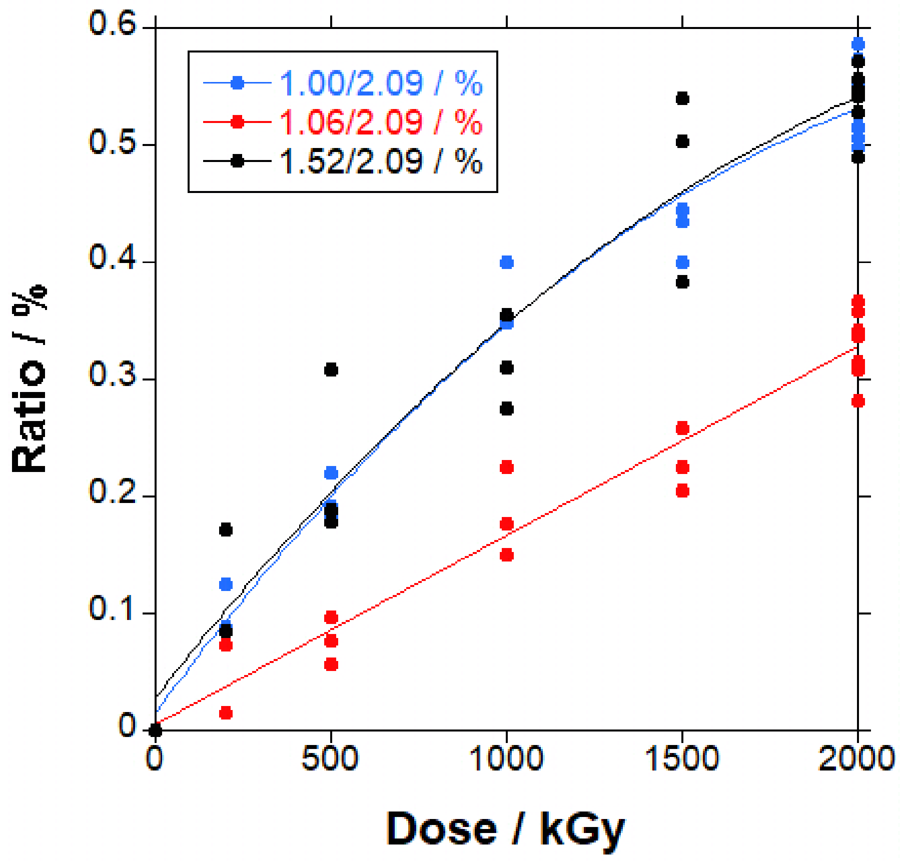

Figure S14. Mol % of the peaks at 1.00 ppm (blue colour), 1.52 ppm (black colour) and 1.06 ppm (red colour) respect to the reference peak for protons 2 at 2.09 ppm. Adjusted lines for peaks 1.00 and 1.52 (quadratic) are just a guide to the eye.

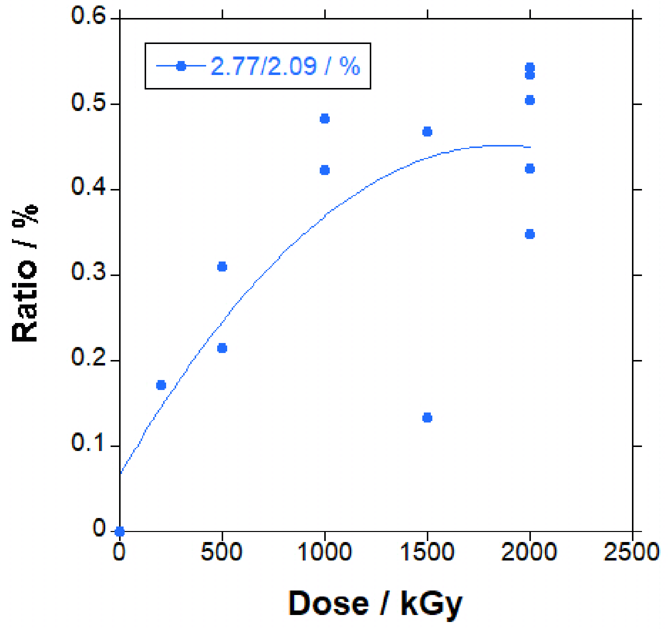

Figure S15. Mol % of the peak at 2.77 ppm respect to the reference peak for protons 2 at 2.09 ppm. Adjusted line (quadratic) is just a guide to the eye

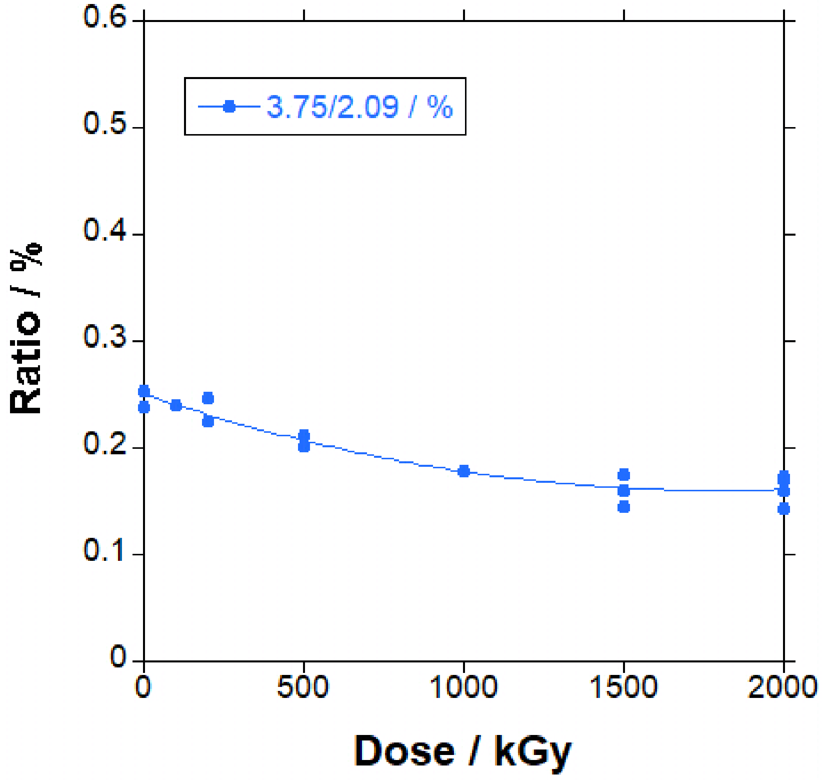

Figure S16. Mol % of the peak at 3.75 ppm respect to the reference peak for protons 2 at 2.09 ppm. Adjusted line (quadratic) is just a guide to the eye

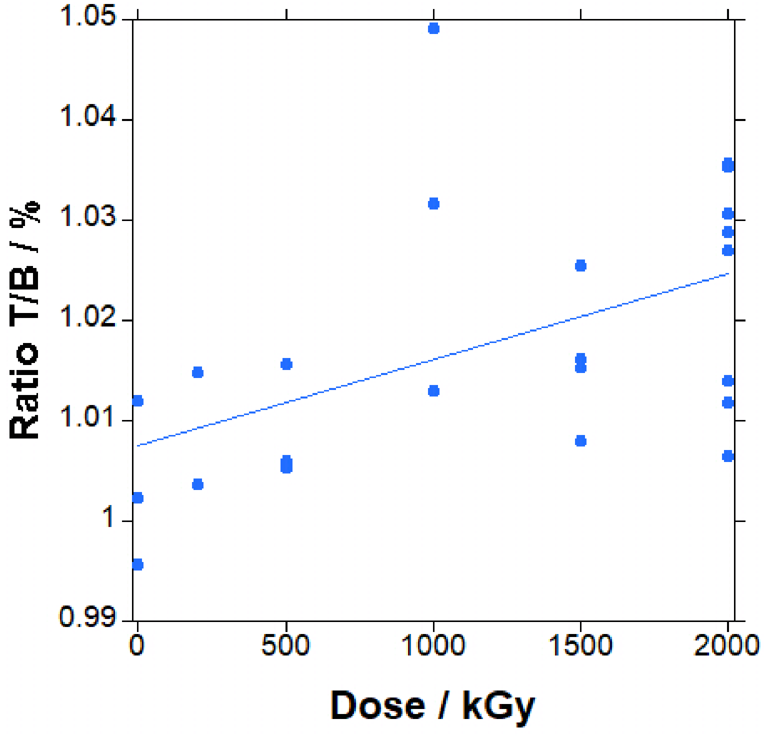

Figure S17. Ratio or aromatic protons (T) to butylene (B) protons

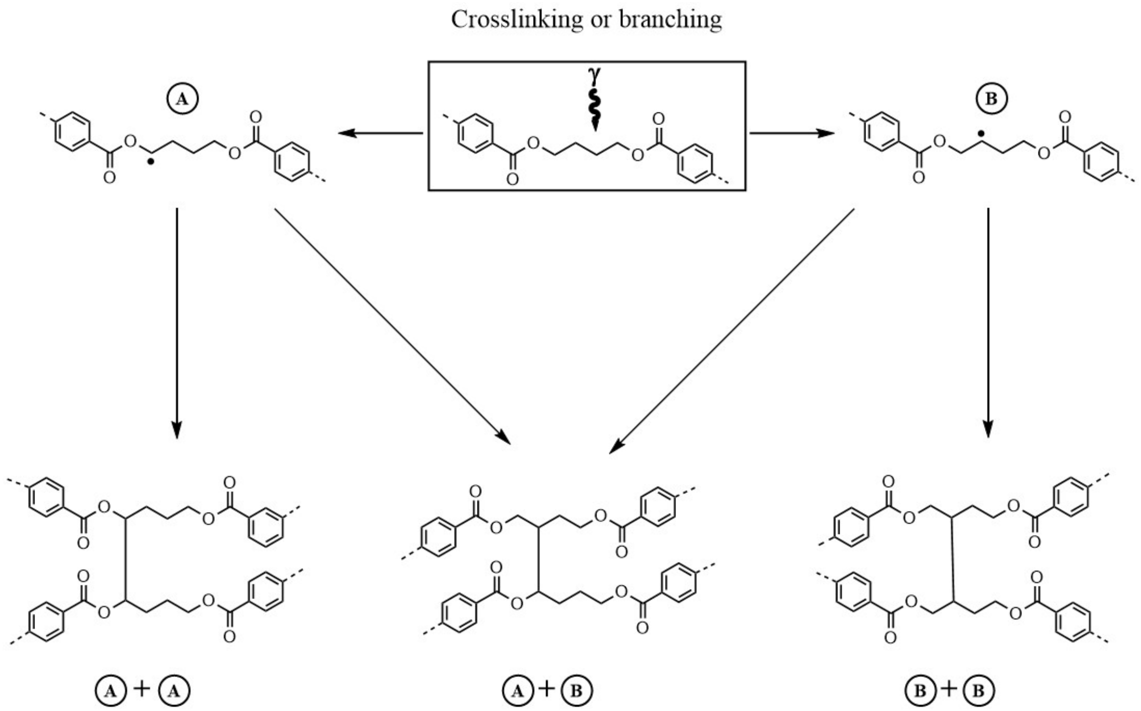

Figure S18. Possible species formed by crosslinking of radicals produced in PBT main chain

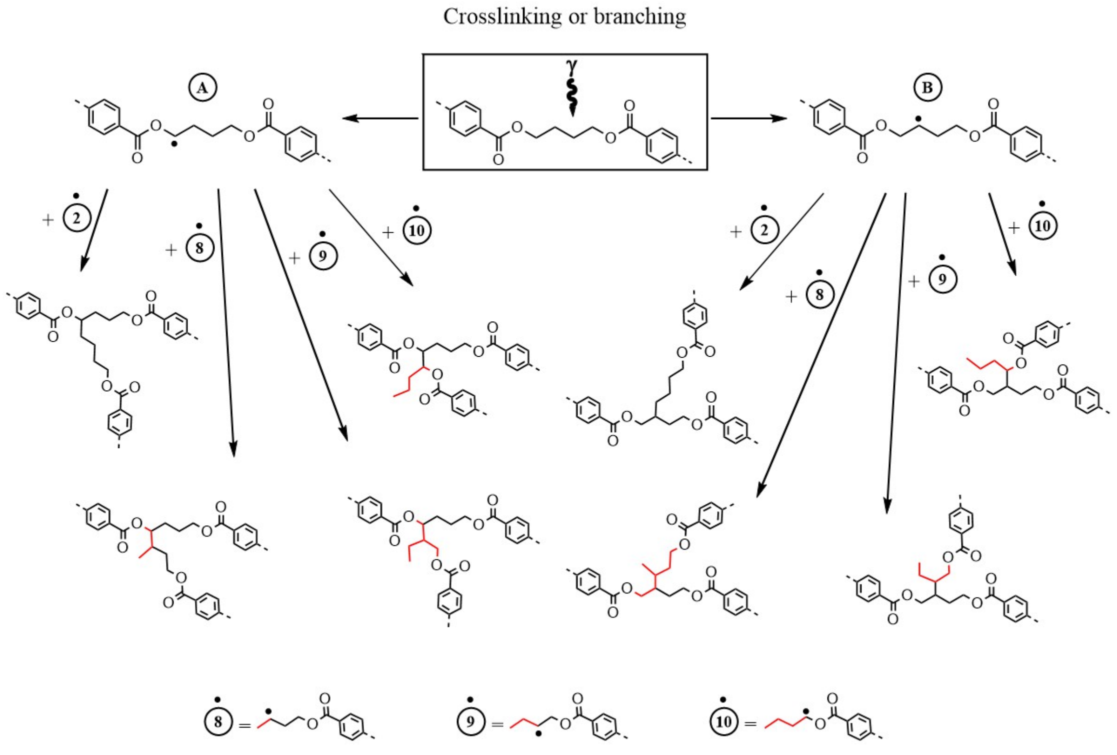

Figure S19. Possible species formed by crosslinking of radicals produced in PBT main chain with radicals produced in the butyl chain ends

Table S1. Calorimetric properties for irradiated PBT – 1 mm thick specimens

Dose (kGy)	Tg_1 (°C)	Tc_1 (°C)	Mp_1 (°C)	ΔH_1 (J/g)	χ_1 (%)	Tc (°C)	ΔHc (J/g)	χ_c (%)	Tg_2 (°C)	Mp'2 (°C)	Mp_2 (°C)	ΔH_2 (J/g)	χ_2 (%)
0	44.4	203.7	225.0	43.23	29.8	188.1	49.56	34.2	40.8	216.6	225.5	54.13	37.3
0	42.6	210.7	224.7	44.67	30.8	186.3	47.16	32.5	40.4	216.1	225.4	52.95	36.5
10	44.2	204.5	226.4	41.22	28.4	187.6	48.08	33.2	42.9	217.2	226.4	51.06	35.2
20	44.5	204.7	226.0	41.10	28.3	187.3	48.03	33.1	44.0	216.9	226.4	51.59	35.6
30	44.1	203.9	225.9	41.38	28.5	187.1	48.15	33.2	42.9	215.8	225.5	51.47	35.5
50	43.8	204.1	225.4	42.74	29.5	186.1	47.78	33.0	42.7	215.7	226.2	51.98	35.8
100	43.8	203.9	225.7	42.20	29.1	187.3	48.43	33.4	41.6	216.1	226.0	52.41	36.1
200	43.9	202.6	225.4	43.06	29.7	186.6	48.70	33.6	42.7	215.0	226.2	53.23	36.7
500	44.9	204.0	226.7	46.81	32.3	188.5	50.02	34.5	44.0	216.9	227.5	55.76	38.5
500	43.5	203.0	226.2	44.51	30.7	188.1	48.70	33.6	40.5	214.7	225.9	54.05	37.3
700	45.3	202.8				188.5	49.20	33.9	42.7	215.5	226.0	53.61	37.0
1000	44.5	201.2	224.4	46.53	32.1	188.8	50.80	35.0	42.0	214.5	224.5	57.12	39.4
1200		201.1	223.5	46.41	32.0	188.8	50.40	34.8	41.1	213.8	223.7	59.28	40.9
1500		199.2	222.9	46.40	32.0	187.6	51.00	35.2	46.0	212.6	223.0	61.36	42.3
2000	42.1	198.6	221.3	47.68	32.9	187.8	52.05	35.9	46.6	211.0	222.5	64.11	44.2

Mp'2 corresponds to the secondary maximum of the melting endotherm on the second heating step

Table S2. Calorimetric properties for irradiated PBT – 1.5 mm thick specimens

Dose (kGy)	Tg_1 (°C)	Tc_1 (°C)	Mp_1 (°C)	ΔH_1 (J/g)	χ_1 (%)	Tc (°C)	ΔHc (J/g)	χ_c (%)	Tg_2 (°C)	Mp'2 (°C)	Mp_2 (°C)	ΔH_2 (J/g)	χ_2 (%)
0	46.3	206.1	226.0	41.20	28.4	186.5	48.63	33.5	41.8	216.5	225.9	53.65	37.0
0	41.7	210.8	224.9	42.99	29.7	186.3	47.15	32.5	40.4	216.0	225.4	52.81	36.4
10	45.3	205.3	225.5	41.56	28.7	186.8	47.09	32.5	39.5	215.9	225.4	52.85	36.4
10	44.0	205.6	225.2	41.40	28.6	187.6	47.62	32.8	43.1	216.1	226.0	54.21	37.4
20	45.8	205.4	224.9	42.04	29.0	186.5	48.32	33.3	41.9	216.1	225.5	53.44	36.9
30	46.1	204.4	225.4	42.33	29.2	186.5	47.03	32.4	41.6	215.1	224.9	53.33	36.8
50	46.5	204.9	225.5	41.38	28.5	187.3	48.35	33.3	41.8	215.8	226.0	52.17	36.0
100	46.1	204.5	225.4	42.00	29.0	188.1	48.72	33.6	42.2	215.7	225.5	52.80	36.4
100	44.7	204.7	225.4	41.11	28.4	188.0	48.57	33.5	41.6	215.6	225.9	51.97	35.8
200	44.5	204.3	225.2	42.95	29.6	187.8	48.97	33.8	39.3	214.4	225.2	53.96	37.2
500	43.5	203.6	223.7	45.28	31.2	188.0	49.75	34.3	40.2	214.4	224.2	54.25	37.4
700	42.9	204.0	224.0	45.05	31.1	188.7	49.62	34.2	40.9	215.6	224.9	55.45	38.2
1000	43.6	201.6	224.2	45.05	31.1	189.3	51.42	35.5	38.6	213.7	224.7	56.12	38.7
1200	42.9	201.0	223.4	46.06	31.8	188.6	50.68	35.0	40.4	212.7	223.9	56.35	38.9
1500	43.8	200.2	223.3	45.74	31.5	188.1	51.17	35.3	42.0	213.6	224.4	56.67	39.1
2000	41.1	200.6	223.6	46.01	31.7	187.6	50.01	34.5	39.2	213.4	223.9	54.55	37.6

Mp'2 corresponds to the secondary maximum of the melting endotherm on the second heating step

Table S3. Calorimetric properties for irradiated PBT – 2 mm thick specimens

Dose (kGy)	Tg_1 (°C)	Tc_1 (°C)	Mp_1 (°C)	ΔH_1 (J/g)	χ_1 (%)	Tc (°C)	ΔHc (J/g)	χ_c (%)	Tg_2 (°C)	Mp’2 (°C)	Mp_2 (°C)	ΔH_2 (J/g)	χ_2 (%)
0	47.9	208.8	229.1	40.79	28.1	188.5	49.15	33.9	42.4	219.4	229.2	53.58	36.9
0	43.2		227.7			188.6	48.59	33.5	41.6	218.6	228.9	53.71	37.0
10	43.6	207.0	226.7	40.45	27.9	187.8	48.26	33.3	42.2	217.4	227.2	51.37	35.4
20	47.8	207.1	226.5	42.67	29.4	187.0	51.77	35.7	42.4	216.5	227.2	57.27	39.5
30	47.2	207.1	225.7	39.60	27.3	188.1	46.25	31.9	41.7	217.9	226.4	51.06	35.2
50	45.6	210.0	230.7			189.0	47.47	32.7	44.1	218.6	229.2	53.18	36.7
100	45.4	207.6	227.5	43.91	30.3	188.5	48.60	33.5	41.2	217.6	227.7	55.50	38.3
100	43.4	206.8	226.5	42.40	29.2	188.1	48.71	33.6	42.7	216.7	227.0	55.10	38.0
200	44.7	208.4	228.4	45.56	31.4	188.6	49.40	34.1	44.2	218.9	228.5	56.74	39.1
500	42.8	206.0	226.0	45.02	31.0	189.3	50.31	34.7	42.2	215.1	225.7	57.01	39.3
700	44.6	205.6	226.5	45.12	31.1	189.0	50.66	34.9	44.9	216.6	227.7	54.25	37.4
1000	42.9	204.6	225.2	47.26	32.6	189.0	52.70	36.3	43.9	215.1	224.2	57.64	39.8
1200	43.6	206.0	227.5	45.10	31.1	188.6	50.17	34.6	43.9	216.9	227.7	55.53	38.3
1500	44.6	202.9	223.0	47.17	32.5	188.3	49.88	34.4	45.9	213.5	222.5	56.26	38.8
2000	43.7	201.2	221.9	48.32	33.3	188.1	53.62	37.0	43.7	211.9	222.2	58.35	40.2

Mp’2 corresponds to the secondary maximum of the melting endotherm on the second heating step

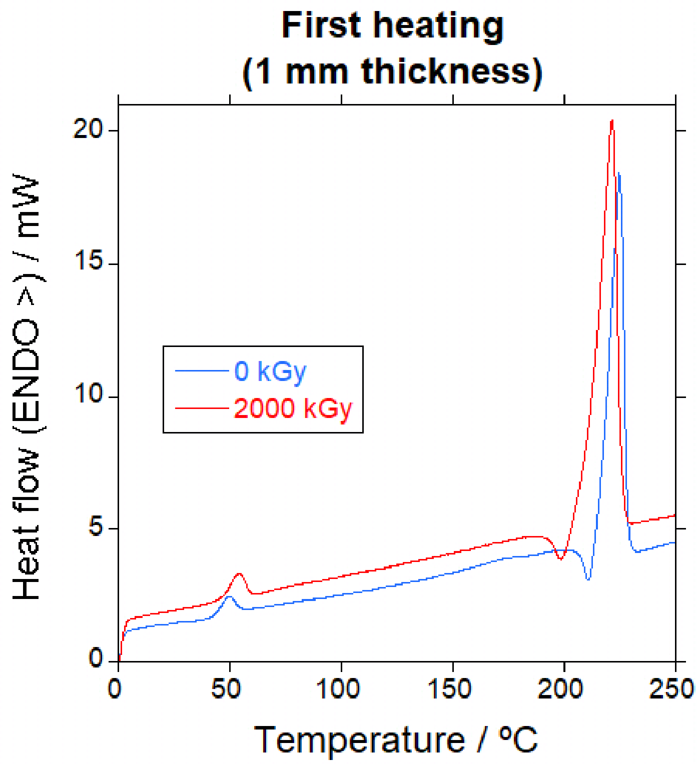

Figure S20. DSC curves in the first heating for unirradiated 1 mm thick PBT (blue colour) and for 1 mm thick PBT irradiated at 2000 kGy (red colour)

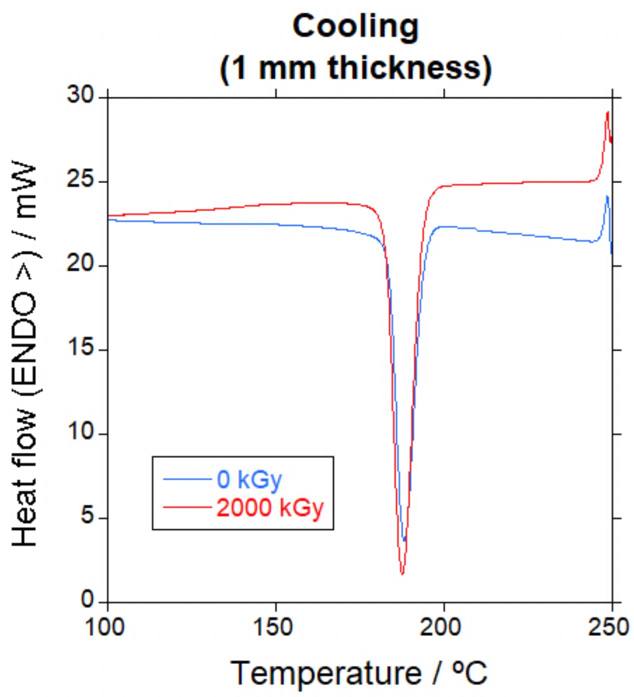

Figure S21. DSC curves in the cooling for unirradiated 1 mm thick PBT (blue colour) and for 1 mm thick PBT irradiated at 2000 kGy (red colour)

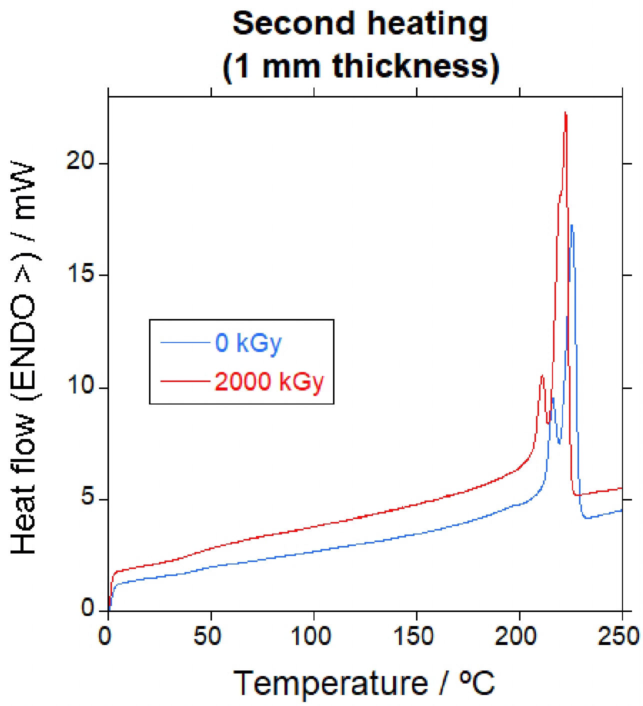

Figure S22. DSC curves in the second heating for unirradiated 1 mm thick PBT (blue colour) and for 1 mm thick PBT irradiated at 2000 kGy (red colour)

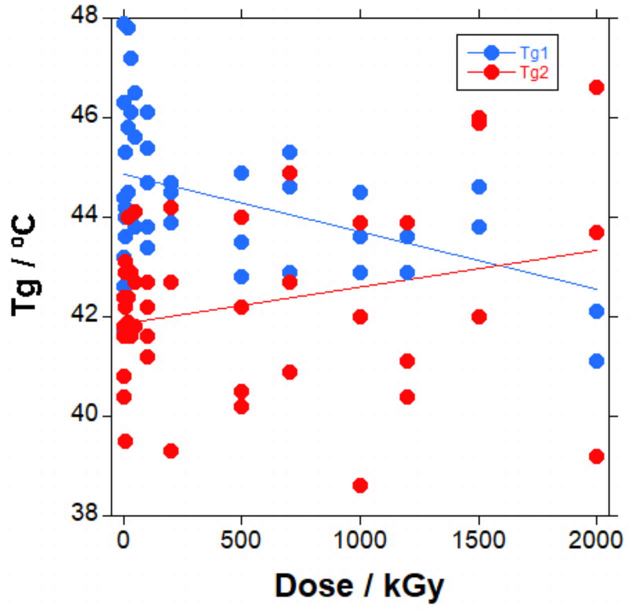

Figure S23. Tg (Tg_1 for the first heating run and Tg_2 for the second heating run) vs dose for irradiated PBT at different doses. Linear fitting of the data is just a guide to the eye.

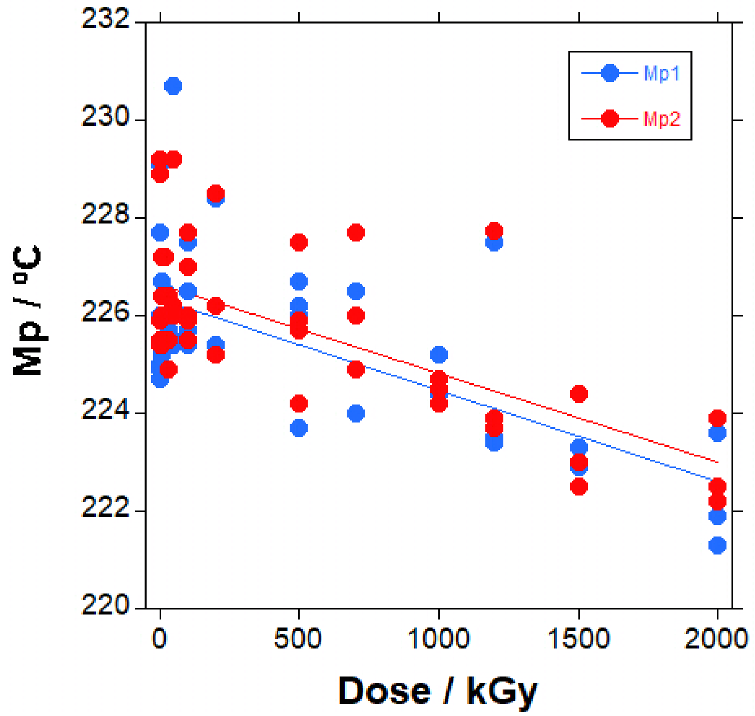

Figure S24. Main melting peak (Mp_1 for the first heating run and Mp_2 for the second heating run for irradiated PBT vs dose.

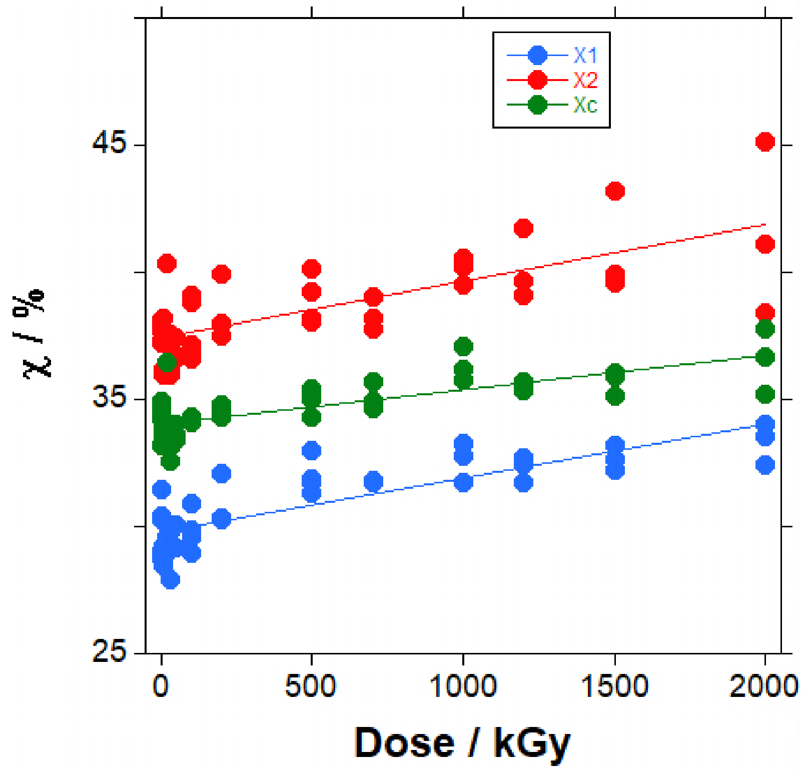

Figure S25. Main melting peak (Mp_1 for the first heating run and Mp_2 for the second heating run for irradiated PBT vs dose.

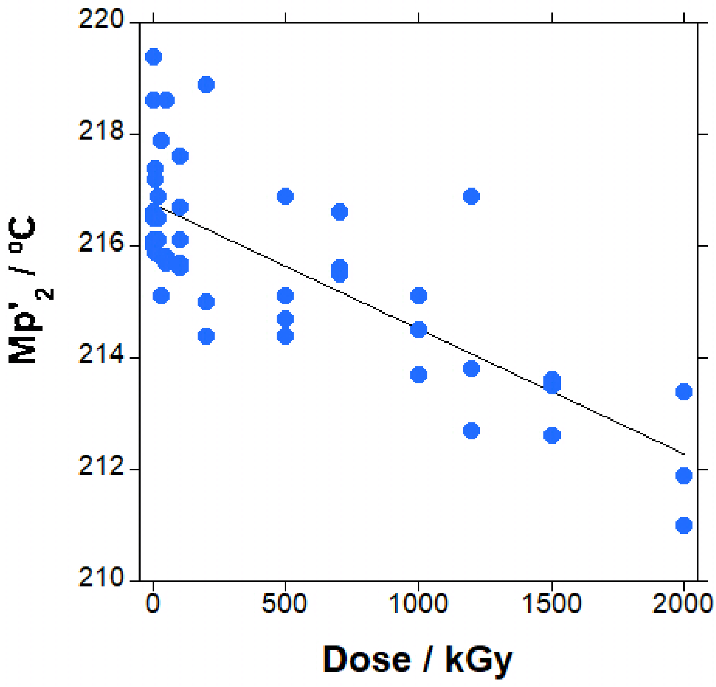

Figure S26. Secondary melting peak (Mp'_2) for the second heating run for irradiated PBT vs dose.

Table S4. Mechanical properties for irradiated PBT – 1 mm thick specimens

Dose / kGy	Stress / MPa	Strain / %	Modulus / MPa
0	67 ± 4	731 ± 15	910 ± 30
10	73 ± 3	770 ± 30	900 ± 50
20	70 ± 6	760 ± 50	870 ± 50
30	69 ± 6	740 ± 40	910 ± 40
50	71.2 ± 1.3	758 ± 16	930 ± 50
100	64 ± 8	700 ± 60	920 ± 40
200	65 ± 5	720 ± 50	950 ± 20
500	58 ± 9	680 ± 80	930 ± 100
700	54 ± 8	680 ± 80	860 ± 70
1000	50.9 ± 0.5	620 ± 30	930 ± 80
1200	37 ± 6	440 ± 90	920 ± 40
1500	29.0 ± 0.6	120 ± 50	880 ± 90
2000	25.7 ± 0.9	24 ± 6	970 ± 40
700	54 ± 8	680 ± 80	860 ± 70

Table S5. Mechanical properties for irradiated PBT – 1.5 mm thick specimens

Dose / kGy	Stress / MPa	Strain / %	Modulus / MPa
0	66 ± 5	720 ± 40	900 ± 40
10	63 ± 3	690 ± 30	910 ± 40
20	61 ± 13	660 ± 120	890 ± 70
30	55 ± 10	610 ± 100	930 ± 50
50	61 ± 10	680 ± 100	900 ± 80
100	54 ± 6	620 ± 60	910 ± 40
100	54 ± 11	650 ± 110	880 ± 30
200	57 ± 11	650 ± 120	890 ± 60
500	51 ± 5	620 ± 50	900 ± 50
700	41 ± 6	500 ± 90	880 ± 30
1000	38.1 ± 1.7	450 ± 30	940 ± 30
1200	32 ± 4	240 ± 160	900 ± 20
1500	28.4 ± 0.6	76 ± 20	860 ± 30
2000	26.0 ± 1.7	26.2 ± 1.7	950 ± 50

Table S6. Mechanical properties for irradiated PBT – 2 mm thick specimens

Dose / kGy	Stress / MPa	Strain / %	Modulus / MPa
0	66 ± 5	720 ± 40	900 ± 40
10	63 ± 3	690 ± 30	910 ± 40
20	61 ± 13	660 ± 120	890 ± 70
30	55 ± 10	610 ± 100	930 ± 50
50	61 ± 10	680 ± 100	900 ± 80
100	54 ± 6	620 ± 60	910 ± 40
100	54 ± 11	650 ± 110	880 ± 30
200	57 ± 11	650 ± 120	890 ± 60
500	51 ± 5	620 ± 50	900 ± 50
700	41 ± 6	500 ± 90	880 ± 30
1000	38.1 ± 1.7	450 ± 30	940 ± 30
1200	32 ± 4	240 ± 160	900 ± 20
1500	28.4 ± 0.6	76 ± 20	860 ± 30
2000	26.0 ± 1.7	26.2 ± 1.7	950 ± 50

Table S7. Mechanical properties for irradiated PBT pellets subsequently injected– 1 mm thick specimens

Dose / kGy	Stress / MPa	Strain / %	Modulus / MPa
0	69 ± 2	768 ± 15	740 ± 40
100	65 ± 3	750 ± 20	750 ± 30
500	52 ± 3	640 ± 40	780 ± 40
1000	40 ± 3	450 ± 60	890 ± 30
1200	36 ± 3	380 ± 80	900 ± 40
1500	33.2 ± 0.5	250 ± 80	870 ± 30
2000	40 ± 30	7 ± 10	873 ± 15

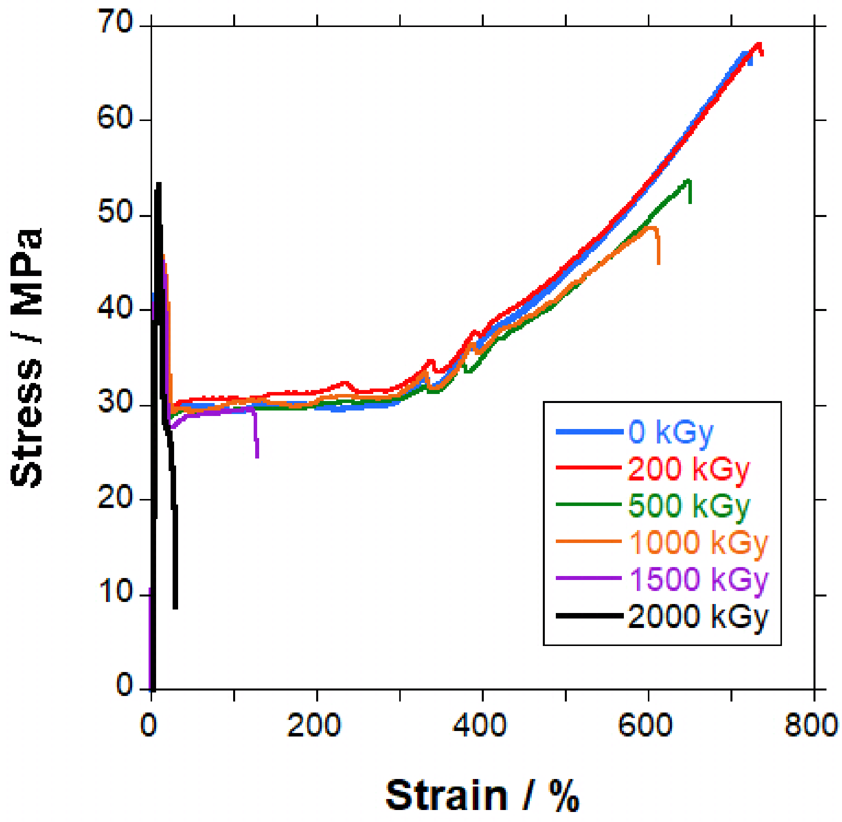

Figure S27. Stress-strain curves for PBT irradiated at different doses

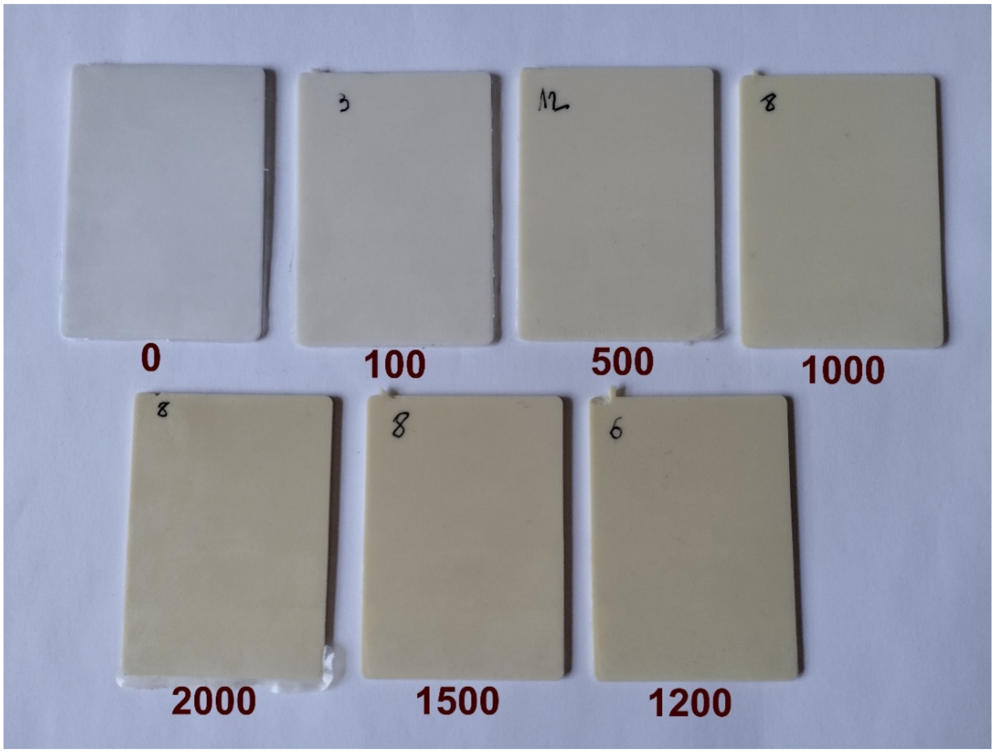

Figure S28. Injected plates from PBT pellets irradiated at different doses

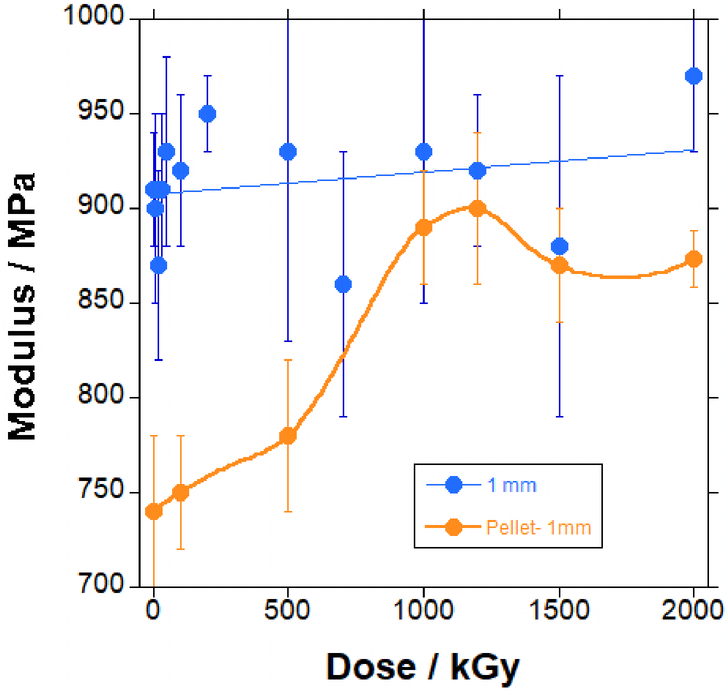

Figure S29. Young's Modulus vs. dose for irradiated PBT dumbbell specimens 1 mm thick (1 mm) and injected irradiated PBT pellets (Pellet 1 mm)

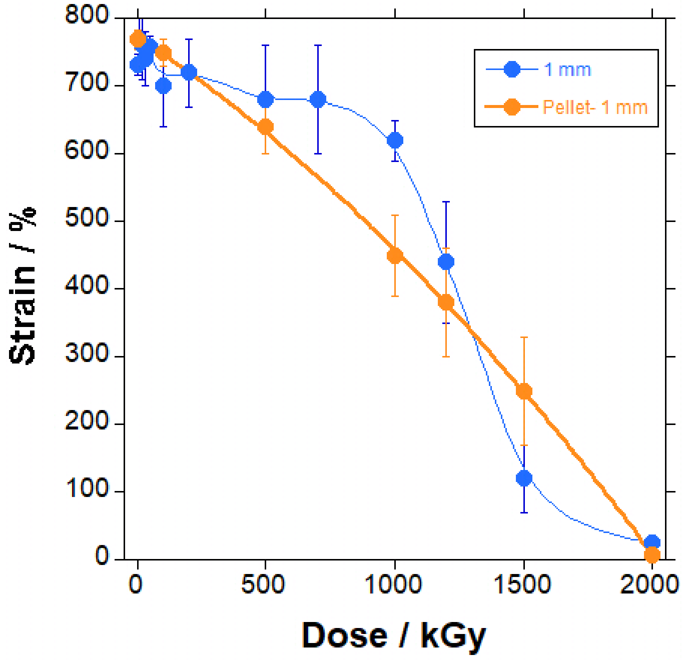

Figure S30. Elongation at break vs. dose for irradiated PBT dumbbell specimens 1 mm thick (1 mm) and injected irradiated PBT pellets (Pellet 1 mm)

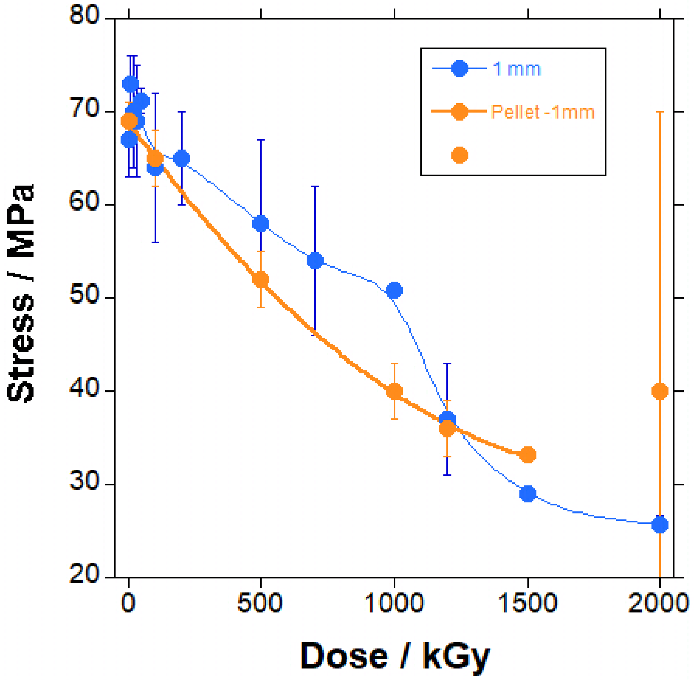

Figure S31. Tensile strength vs. dose for irradiated PBT dumbbell specimens 1 mm thick (1 mm) and injected irradiated PBT pellets (Pellet 1 mm)

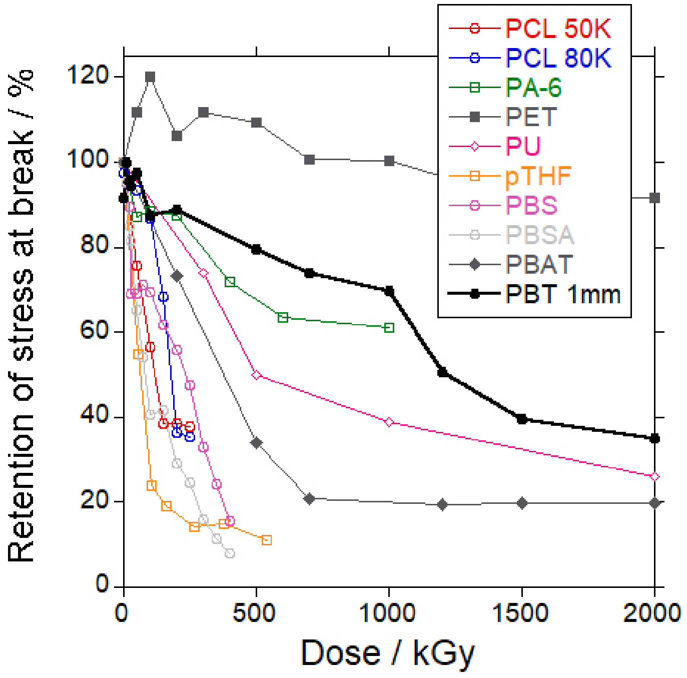

Figure S32. Retention of tensile strength vs dose for PBT (black filled circles), PBAT (dark grey filled diamonds), PBS (purple circles), PBSA (grey circles), PCL 50K (red circles), PCL 80K (blue circles), PA-6 (green squares), PET (dark grey filled squares), aliphatic polyurethane (magenta diamonds) and poly(tetramethylene oxide) (orange squares).

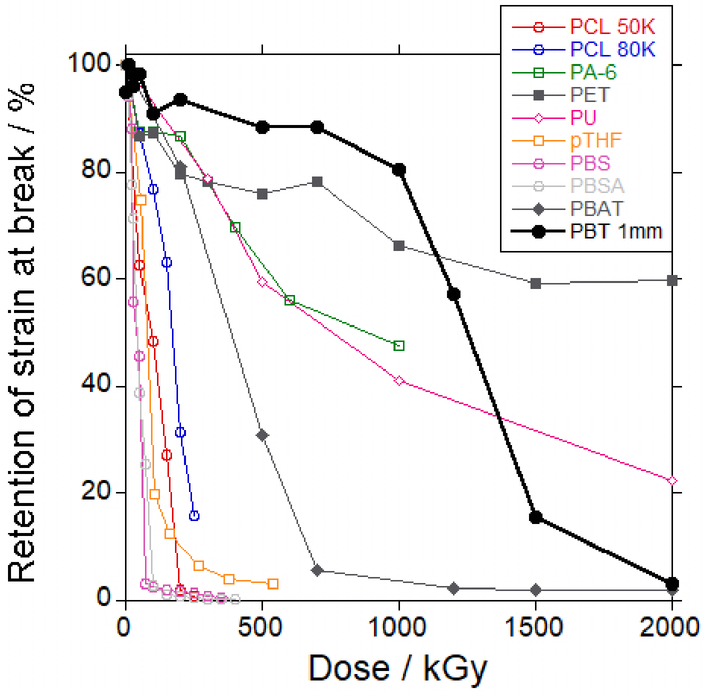

Figure S33. Retention of tensile strain vs dose for PBT (black filled circles), PBAT (dark grey filled diamonds), PBS (purple circles), PBSA (grey circles), PCL 50K (red circles), PCL 80K (blue circles), PA-6 (green squares), PET (dark grey filled squares), aliphatic polyurethane (magenta diamonds) and poly(tetramethylene oxide) (orange squares).
